# A scalable data driven geospatial framework for climate risk assessment

**DOI:** 10.1038/s41598-025-32370-7

**Published:** 2025-12-20

**Authors:** Moustafa Naiem Abdel-Mooty, Paulin Coulibaly, Wael El-Dakhakhni

**Affiliations:** 1 Department of Civil Engineering, McMaster University, 1280 Main Street West, Hamilton, ON L8S 4L7 Canada; 2 NSERC FloodNet, Department of Civil Engineering, McMaster University, 1280 Main Street West, Hamilton, ON L8S 4L7 Canada; 3 Department of Civil Engineering and School of Computational Science and Engineering, McMaster University, 1280 Main Street West, Hamilton, ON L8S 4L7 Canada; 4 RESILIOCS Intelligence, Hamilton, Canada

**Keywords:** Interpretability, Climate impact, Flood hazard, Climate risk, Machine learning, Resilience, Climate Adaptation, Digital twin, Climate change, Civil engineering

## Abstract

Traditional flood risk management approaches often rely on historical data, limiting their ability to account for the increasing severity and frequency of climate-induced hazards. This study presents a scalable, data-driven framework that integrates geospatial analysis and machine learning to dynamically assess climate risks. The framework enables decision-makers to identify vulnerabilities, quantify flood risk under evolving climate scenarios, and develop informed adaptation strategies. Using bias-corrected CMIP5 climate projections as use case, the framework is demonstrated through a case study in Texas, where community flood risk prediction is done under multiple emission scenarios. Results indicate that under RCP 8.5, community vulnerability is projected to increase by 14%, leading to an estimated 28% rise in economic damages ($1.8B per decade by 2050) and heightened socio-economic disruptions, including displacement and infrastructure failures. By identifying the most influential climatological factors that impact community resilience, our approach stresses the urgent need for global action to mitigate extreme climate scenarios. It shows the scalability and flexibility of the framework, emphasizing its potential as decision-support tool, and a step towards a digital twin system for climate risk assessment and adaptation planning.

**Supplementary Information:**

The online version contains supplementary material available at 10.1038/s41598-025-32370-7.

## Introduction

Over the past three decades, the magnitude and frequency of climate-induced disasters (e.g., climatological and hydrological) have been increasing at an alarming rate, jeopardizing the livelihoods of millions living in at-risk communities^1–3^. However, most current methodologies adopted for flood risk management assume that historical data serves as a good predictor for future projections in its current trajectory^4^. The weather conditions have been heavily impacted by the changing climatological conditions (i.e., air humidity, precipitation, and temperature). The data indicates that North America is experiencing a rise in extreme rainfall events, combined with increasing urbanization, resulting in more flooding incidents^5–7^. This is exacerbated by the increased urbanization and population growth into flood-prone areas, where it is expected that by the year 2050, 70% of the world population will be inhabiting urban environments, in contrast to the 2020 figure of 50%^8,9^. As climate-induced hazards grow in frequency and severity, adaptive planning strategies must evolve to integrate real-time, data-driven decision-making frameworks^10,11^. In this context, Digital Twin technology has emerged as a transformative tool, enabling simulation-based modeling and predictive analytics for asset management and climate resilience. However, existing Digital Twin applications typically focus on localized, asset-level representations, limiting their ability to capture broader spatial, environmental, and socio-economic dynamics that influence resilience planning at a systemic level^12^.

To address this limitation, this study introduces a scalable, data driven geospatial framework that draws inspiration from Digital Twin principles while remaining distinct from full digital twin systems. Unlike traditional Digital Twins that depend on real time sensing and continuous synchronization with physical assets, this framework leverages large scale geospatial, climatic, and socio environmental datasets to generate scalable, data driven insights for climate risk assessment and adaptation decision support. Unlike conventional Digital Twins, which primarily model individual assets or infrastructure components, the current framework is designed to provide a system-wide perspective, allowing policymakers, urban planners, and emergency management agencies to assess cascading climate risks, anticipate vulnerabilities, and implement targeted adaptation strategies. The novelty of the framework lies in its ability to synthesize diverse datasets, including historical climate records, land-use patterns, hazard exposure data, and resilience indicators (explicit and implicit), into a scalable, spatially explicit model. This enables a more comprehensive approach to resilience planning, ensuring that decision-makers can account for both localized impacts and regional interdependencies.

To achieve this goal, it is essential to define resilience and vulnerability in the context of the current study, enabling the framework to be applied in various settings. Defined by exposure, sensitivity, and adaptive capacity, *vulnerability* sets the stage for transformative change within communities, acting to identify areas in need of improvement^13^. Sustainability, including environmental, social, and economic dimensions, emerges as a key mitigator of vulnerability. Sustainable practices, encompassing Environmental stewardship, economic viability, responsible governance, resilience and adaptability, just resource management, social equity serve as buffers against various stressors, creating a foundation for resilience^14,15^. Resilience, in turn, becomes both an outcome of sustainable practices and a response to vulnerability. Communities engaged in sustainable development cultivate resilience, leveraging sustainable resource utilization, inclusive social structures, and effective governance to withstand and recover from external stressors, whether anthropogenic or natural. This interdependent nature between sustainability, vulnerability, and resilience forms a closed loop where each aspect needs to be identified, examined, and addressed^16^. It is essential to recognize that planning for resilient cities is a crucial component of urban and city planning. However, due to the complexity of existing and emerging risks facing urban communities, such as interconnected climate-induced hazards, infrastructure vulnerabilities, growing urbanization, and the rising severity of climate risks, it is crucial to strengthen overall community resilience to ensure life is sustained, societies are supported, and risks are mitigated and vulnerabilities are addressed. Recognizing vulnerability as a precursor to change, leveraging sustainability as a tool for mitigating vulnerabilities, and understanding resilience as the outcome of sustainable practices are crucial for communities to navigate the evolving challenges and forge paths towards adaptive, sustainable, and resilient futures^13,17–19^.

In this manuscript, resilience is defined as the ability of a community to resist the effects of a realized flood risk, and rapidly recover from the former to its pre-event, or other target, functionality^20,21^. Resilience is thus characterized by its two key goals: *Robustness*, the inherent capacity of the system to withstand the effects of an external disruptive event without loss of functionality, and *Rapidity*, the ability of a system to recover to its pre-event levels in a timely manner. These goals are enabled by the resilience means: *Resourcefulness*, the available resources at the system’s disposal to allocate for a rapid recovery, and *Redundancie*s, the inherent system’s replacements (i.e., alternative resources) for adaptive behaviour for functionality retention during a disruptive event^22^.

While progress has been made in employing physics based (hydrological hydraulic) models to map flood resilience^4,23–25^, these models depend on mechanistic equations and boundary conditions tailored to specific systems, which makes them computationally intensive and spatially constrained^24–26^. Conversely, the data driven approach adopted in this study leverages large, heterogeneous climate and impact datasets to identify resilience patterns across broader spatial scales. The two approaches are therefore complementary: physics-based models excel at local scale process representation, whereas the present framework provides scalable insights suitable for regional and national-scale planning.

This study presents the development of a data-driven geospatial framework and demonstrates its usefulness through a case study in Texas. The research objectives are threefold: (1) to design a method for integrating geospatial and climate data into a decision-support system, (2) to test its effectiveness in a real-world case study, and (3) to assess its potential to support climate resilience planning at different levels of governance. By advancing a data-driven, spatially adaptive framework, this research contributes to climate adaptation efforts by providing a structured approach to improving resilience assessments, risk modelling, and policy decision-making. The results highlight the feasibility of deploying this framework on a large scale, providing a transformative tool for climate risk assessment in urban and regional settings.

The framework is designed to enhance resilience-guided flood risk management by integrating climate change effects into community resilience studies. Specifically, it:

- Bypasses the complexity of physics-based modeling by leveraging direct data-driven techniques.
- Projects the effects of climate change on community resilience, considering multiple emission scenarios.
- Explicitly and implicitly incorporates resilience attributes (e.g., robustness and rapidity) alongside community vulnerability and hazard exposure.”

**Fig. 1:**
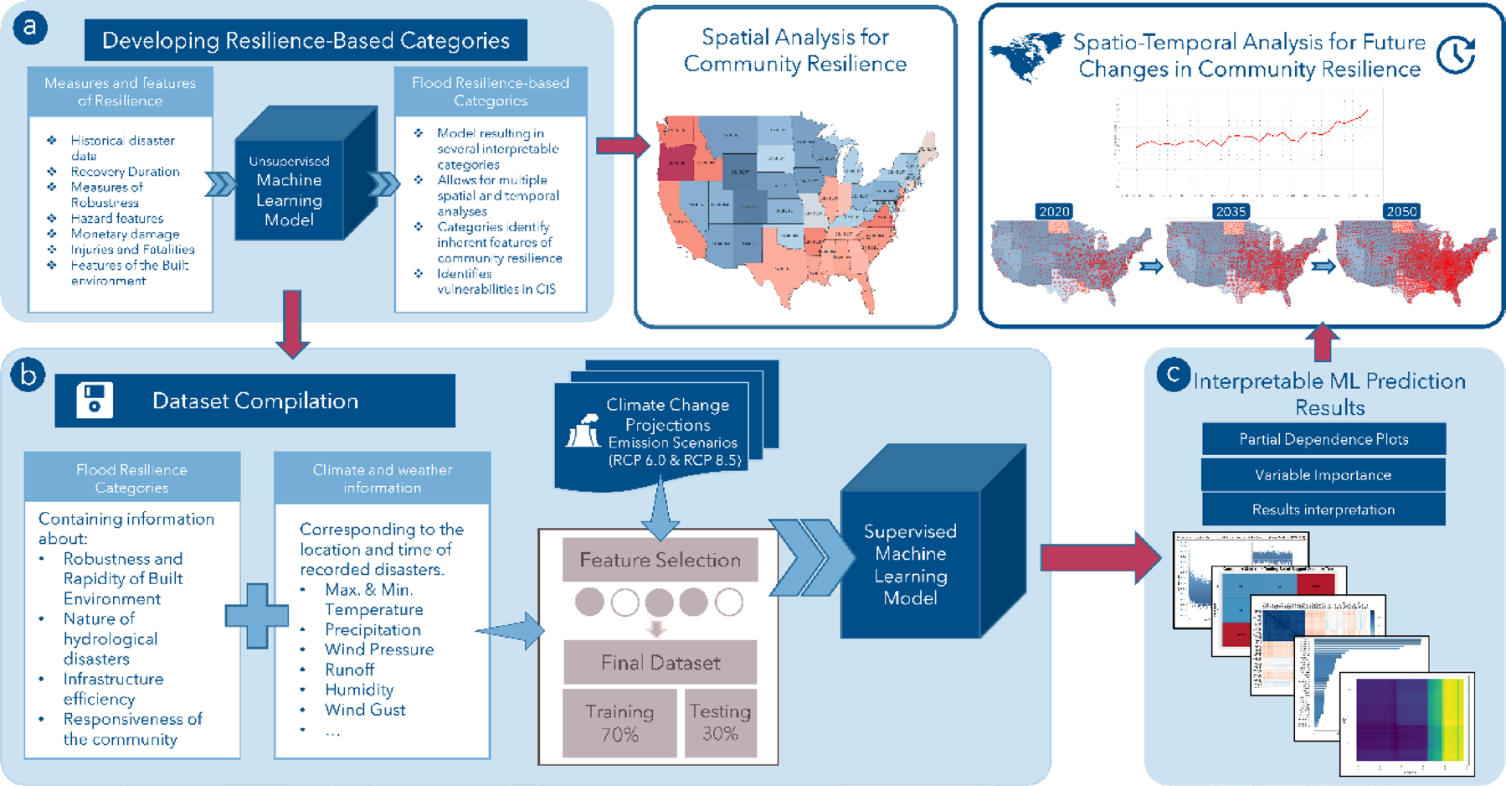
Framework for developing the Scalable geospatial framework for Data-Driven Climate Resilience in Urban Flooding.

However, as with all data-driven techniques, the framework’s effectiveness relies on the quality and diversity of available data, emphasizing the need for comprehensive datasets spanning multiple years. The adopted methodology follows a three-step data-centric approach, as summarized in Fig. 1:

- Step (a) - Resilience-Based Community Classification:

This step employs unsupervised machine learning (clustering techniques) to classify communities based on resilience indicators. These categories serve as the basis for subsequent analyses. Before moving to Step (b), the classified communities undergo spatial analysis to identify the most vulnerable and exposed regions at a regional level, guiding resilience-based decision-making and testbed selection.

- Step (b) - Climate-Resilience Integration:

The community resilience categories from Step (a) are combined with climate data, including vulnerability, exposure, and resilience attributes alongside Global Climate Models (GCMs). This step evaluates climate change effects on resilience paths and considers multiple emission scenarios to assess the long-term impacts of climate risks and possible intervention approaches.

- Step (c) - Model Interpretation and Spatio-Temporal Analysis:

Supervised machine learning models are evaluated using interpretability techniques to identify key resilience drivers and their influence on future predictions. This process aids in spatio-temporal resilience assessments across different scales, supporting data-driven climate adaptation strategies. The approach uses spatiotemporal analytics to model and manage interactions between infrastructure and the environment amid climate change. This framework offers decision-makers a scalable tool for assessing, visualizing, and predicting climate risk across various environmental and socioeconomic conditions. Additional details on each methodological step and the specific algorithms used are included in the following sections. While the current case study is implemented at the county level, the framework is adaptable and can be extended through the integration of high-resolution spatial datasets such as infrastructure, land use, and terrain. Additionally, in this study, the term ‘location-specific’ refers to data resolution at the county level. All feature datasets were obtained at this scale, and no further aggregation or disaggregation was necessary.

## Results

### Resilience categorization and spatial analysis

**Fig. 2:**
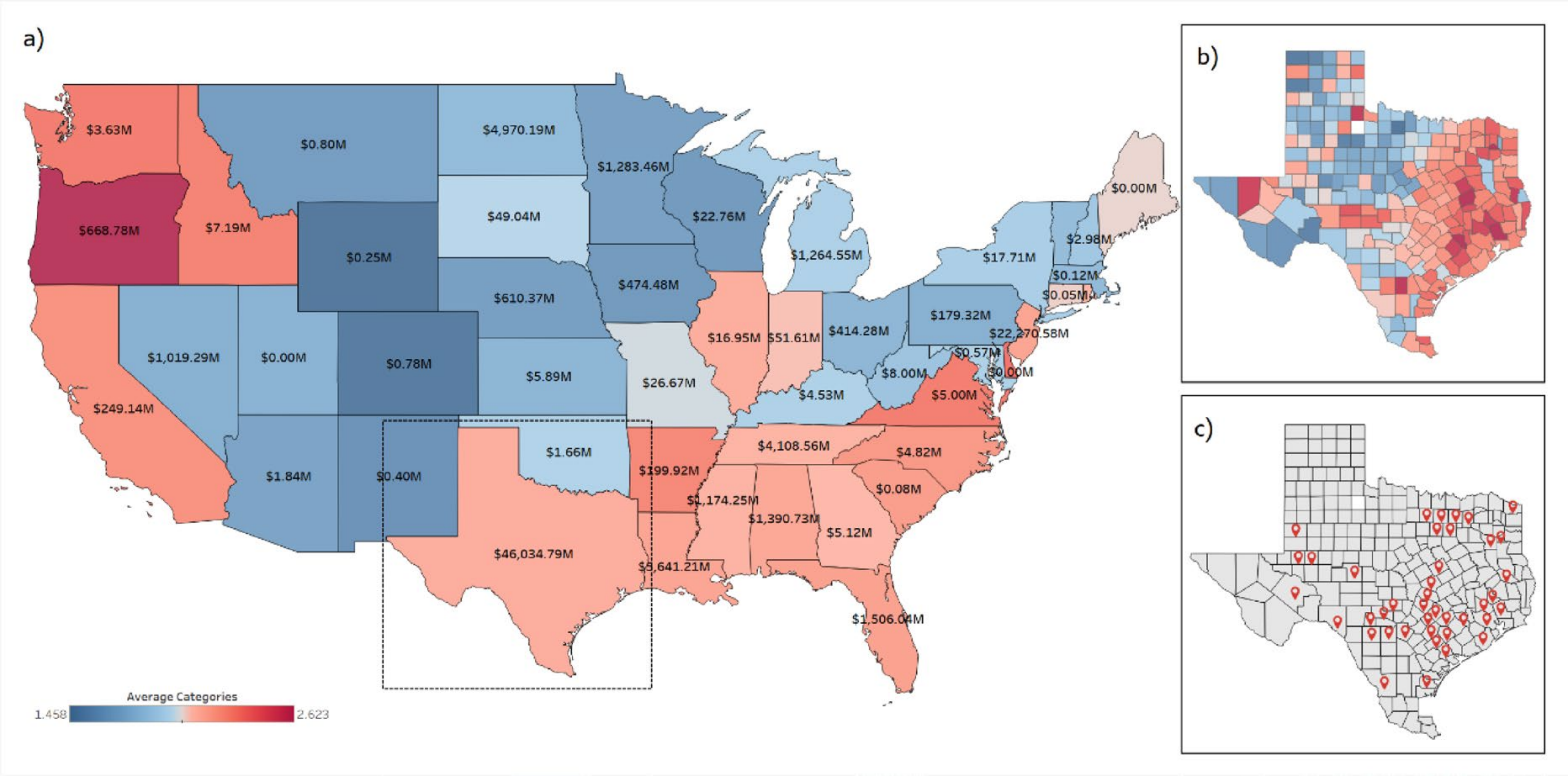
Spatial analysis of the United States showing Monetary Losses and the 3 Categories Flood Vulnerability index, (**a**) Countrywide Spatial analysis at a state level; (**b**) Spatial analysis for the state of Texas at a county level; (**c**) location of collection stations for the climate projections and the employed GCMs. These are Author-generated choropleth maps of projected damages and resilience classifications. Maps were created by the authors in Tableau Desktop (version 2024.1.1; Tableau Software, LLC; www.tableau.com) using the default Tableau basemap. Basemap data ©OpenStreetMap contributors.

For the categorization stage of this model, the categories present in the study by Abdel-Mooty et al.^27^ were employed. In that study, the historical disaster dataset by the NWS was employed; this dataset is considered the longest run flood disaster damage recorded in the United States^28^. The categories developed in that study account for the inherent location-specific characteristics, by including variables that represent the community’s reaction to the considered weather events. This is achieved implicitly by coupling the magnitude of the hazard with the resulting damage, as well as the different variables representing the robustness and rapidity of the specific community where the unsupervised model is applied. These features inherently include information representing numerous aspects of resilience, such as the number of displaced people, injuries, direct damage, and indirect damage (including lost opportunity cost, business losses, displacement costs, crop damage, etc.). These different features also indirectly represent information pertaining to proximity to safe locations, local counter-flood measures in place, and discrepancies in individual risk management plans and policies from one location to another. The data narrative included in the dataset, when available, would contain information regarding the recovery time for each flood disaster. The Rapidity aspect of resilience is the overall recovery time of the community back to its predefined functionality state; however, only the downtime is recorded in the dataset, and the indirect damage was used to imply the recovery duration. The authors acknowledge that this is a limitation of the employed dataset, but given its overall utility, diversity, and variability, it was still sufficient for use in the development of these categories. Those variables were all considered and represented in the developed factors, which represent community resilience through its vulnerability. Such variables are heavily influenced by the regulations and policies established for the areas under consideration. Accordingly, the entire US mainland was included in the development of the resilience categories to capture the variability in how different communities react, given their inherent policies, land use, demographics, and other variables. Noting that the developed categories (indices) increase with the increased loss of resilience, the authors have elected to name it Flood Vulnerability Index (FVI), representing the loss of overall resilience when faced with flood hazards. The categorization process presented in that study resulted in a total of 5 categories. However, further investigation of the employable dataset in the current study with respect to the developed categories revealed clear skewness in the categories, with just 20 observed events falling in category 3, and 102 falling in category 5, representing less than 2% of the dataset, making the inclusion of these categories in developing and training the ML algorithm next to impossible. As such, due to the proximity of the clusters in the unsupervised ML results displayed in Abdel-Mooty et al. (2021), Categories 3, 4, and 5 were merged, resulting in three categories for deployment in the Current study. The resulting categories were used in the development of spatial and descriptive analyses of the disaster database, as shown in Fig. 2, the state of Texas suffered the most damage resulting from flood disaster since the year 1996 and exposed to the highest number of recorded observations, making it appropriate as a testbed for the current study.

**Table 1:** The employed community flood resilience categories.

Community flood vulnerability index	Class description
1	Communities exposed to events that occur in the summer, causing disturbance less than 264 h (11 days) and/or causes up to 250 injuries, and damage less than $2.5B without fatalities
2	Communities exposed to events that occur in the spring, causing any disturbance duration, causes up to 20 injuries, and damage up to $1.5B without fatalities
3	Communities exposed to events occurring in any season, causing any disturbance duration that results in more than 250 injuries, causing damage more than $2.5B, with fatalities, Communities exposed to events that occur in winter or fall, causing disturbance less than 264 h (11 days) causes up to 250 injuries and damage up to $2.5B without fatalities, and Communities exposed to events occurring in the spring that are not under class 2.

Although Texas does not have the highest historical average Flood Vulnerability Index, it still suffered the highest damage at $46B, and a total of 12,834 recorded events. The clear difference between the state of Texas and other states in terms of vulnerability to flood hazards is attributed to the higher heat content associated with the western Gulf of Mexico, which facilitates an increase in humidity and mean temperature compared to other places in the United States^29^. This increased heat content is also proportional to the precipitation resulting from different storms and causes the tropical weather region engulfing the state of Texas, resulting in an alarmingly increasing number of hurricanes and other extreme weather events^30^. This phenomenon is only exacerbated by the increasing urbanization rate, increasing the vulnerable and exposed areas to such extreme weather events^29–31^.

Figure 2a shows the spatial analysis conducted on the historical dataset, showing the monetary damage, and flood resilience category per state as identified in Table 1; Fig. 2b shows a spatial analysis of the state of Texas at a county level, showing a distribution of higher resilience categories along the eastern coast and the Gulf of Mexico. That is attributed to the high-tide flooding and increased sea level, which has become increasingly common over the past decades^32^. The spatial distribution presented in Fig. 2b aligns with the “Cartographic Maps of Precipitation Frequency Estimates” published by NOAA in the report Atlas 14 Volume 11 of Texas^33^, showing an increase in the severity of natural hazards befalling this area (e.g., Hurricane Harvey, with its gigantic $128.8B tally on the coastal communities, identified as one of the most expensive natural disasters in recorded history)^32,34,35^. Figure 2c shows the identified locations where the GCM simulations were run, with 45 counties (listed in Table 2) selected for collecting the CMIP5 simulation results, which were then synchronized with the historical disaster dataset in these counties. It can be observed from Fig. 2b that the vulnerability index varies significantly between counties in proximity (e.g., Hudspeth and Culberson counties). The reason for this is hypothesized such that the change in the category comes from the response of each location to the flood event. Given the proximity of both counties, it is more likely that this discrepancy is due to certain location-specific risk management plans, counter-flood measures, and the implementation of said plans by the occupants. It reflects the overall readiness of the county to face such hazard, which is dependent on the measures and policies put in place. This is further expanded upon in the methodology section.

**Table 2:** List of counties for CMIP5 GCM simulations.

List of Counties were the CMIP5 GCMs Simulation results were extracted						
Bexar	Williamson	Lavaca	Johnson	Bastrop	Real	Bowie
Tarrant	Val Verde	Brazoria	Ellis	Medina	Caldwell	Victoria
Travis	Bell	Liberty	Gaines	Tom Green	Webb	
Dallas	Denton	Angelina	Pecos	Kerr	Hunt	
Nueces	Ector	Montgomery	Austin	McLennan	Collin	
Midland	San Patricio	Smith	Waller	Gonzales	Fayette	
Uvalde	DeWitt	Gregg	San Jacinto	Gillespie	Wise	

The dataset used in developing the prediction algorithm is the Monthly Average Flood Resilience Category, derived by aggregating the average monthly category for each county. This resilience category serves as the dependent variable in the supervised machine learning model, representing community-levels obtained from the earlier unsupervised machine learning step. The independent variables consist of data from CMIP5-simulated GCMs at each of the 45 selected locations: monthly average maximum surface air temperature (°C), monthly average minimum surface air temperature (°C), monthly mean wind speed (m/s), average monthly runoff (mm), and monthly precipitation (mm), for each of the 16 selected GCMs under the selected climate scenario, with a total of 4324 observations. This dataset was split into training and testing datasets at a ratio of 70% and 30%, respectively (3028 observations in the training set and 1296 in the testing set). With results from all GCMs, the total number of input variables is 80 (5 hydrological variables for each of the 16 models). The Brute-Force feature selection method was adopted in this study, rather than an ensemble approach, to avoid introducing aggregation bias and to capture the full variability offered by all models.

**Fig. 3:**
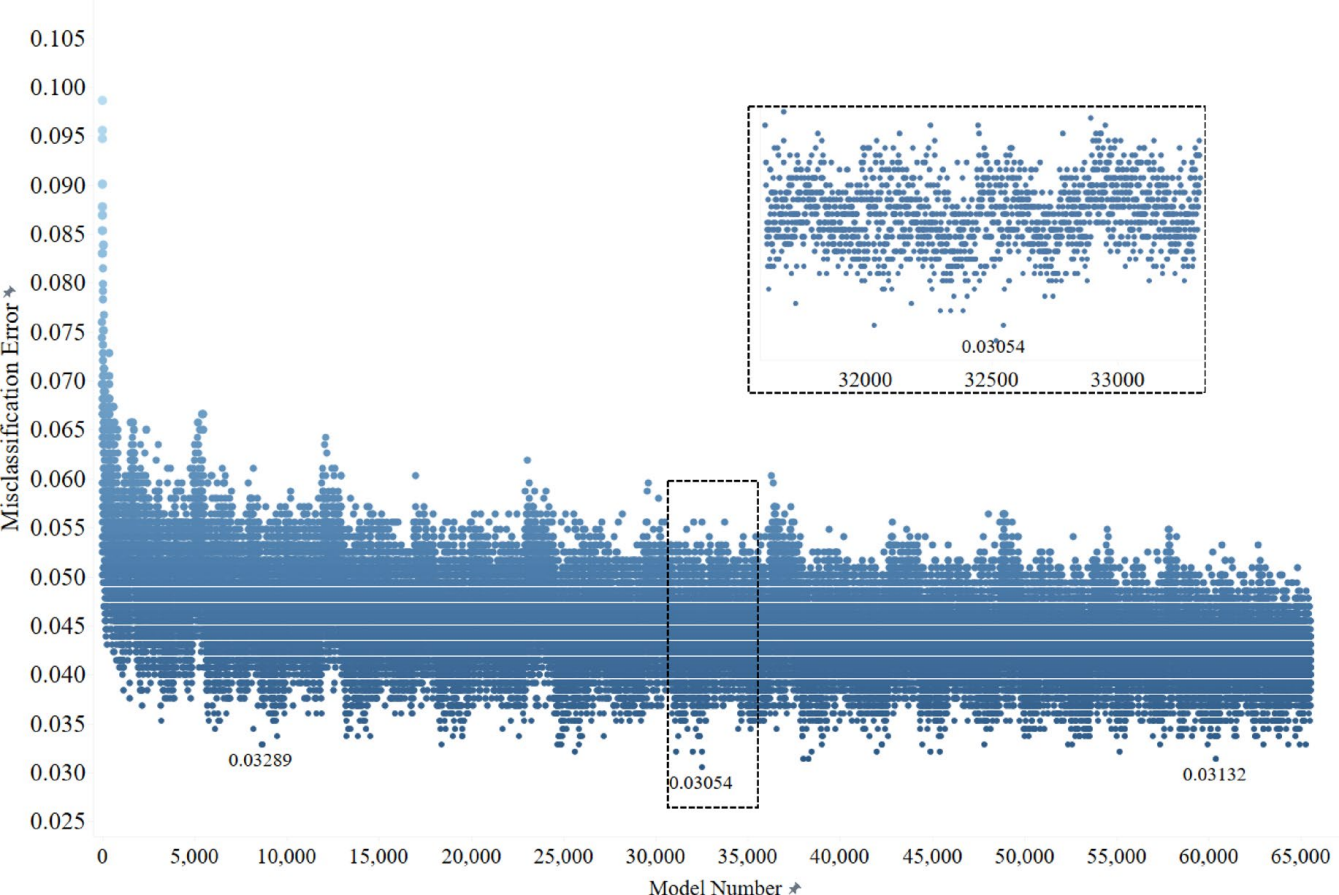
Total misclassification error for all possible GCM combinations for RCP 8.5.

The Brute-Force method relies on the computational capabilities to conduct an exhaustive search throughout all possible combinations of a certain set of variables^36^. The total number of models resulted from all possible combinations for the 16 GCMs for testing were 65,535 models. In this step, a Bagged DT model was used to identify performance measure for all combinations, and the total Misclassification error was selected as the appropriate criteria. Figure 3 shows the misclassification error for all possible combinations for GHG emission scenario RCP 8.5, identifying the model number 59,643 with 11 GCMs included in the analysis (3.1%) (GCMs: 1, 2, 3, 5, 6, 9, 11, 12, 14, 15, 16, with their details in Table 3).

Although the brute-force approach explored an extensive number of potential GCM subsets to identify the combination yielding the lowest misclassification error, we acknowledge that this selection strategy introduces risks of overfitting. The subset optimization was performed using the same dataset which may lead to tuning that reflects the idiosyncrasies of the training data rather than patterns that generalize to unseen conditions. Furthermore, while the procedure was repeated on a county-by-county basis to reduce spatial bias, the selected subsets should not be interpreted as universally optimal. Future applications in different geographic contexts or under evolving climate scenarios would require retraining and reassessment of GCM subsets, as conducted in the current study.

Multiple ML models were developed for predicting the future projection of resilience category across the identified geographical locations within the state of Texas. The first model is a gradient-boosted RF model, with a 70%:30% split of the data, as mentioned earlier, for training and testing, respectively. The model’s performance was optimized with the average Out-of-Bag error (OOB). The second Model is DT with bagging performance enhancement, with 15,000 bootstrap replications, and 4 random splits for variables at each split, and a shrinkage parameter of 0.01 for both emission scenarios. The model’s results were further analyzed with model performance measures and interpretability techniques to choose the optimum model for future projections (i.e., F1-Score, Precision, and Recall (Sensitivity)). This analysis showed that the Bagged DT model had an overall better performance and was thus chosen for the study presented herein.

### Interpretability

**Fig. 4:**
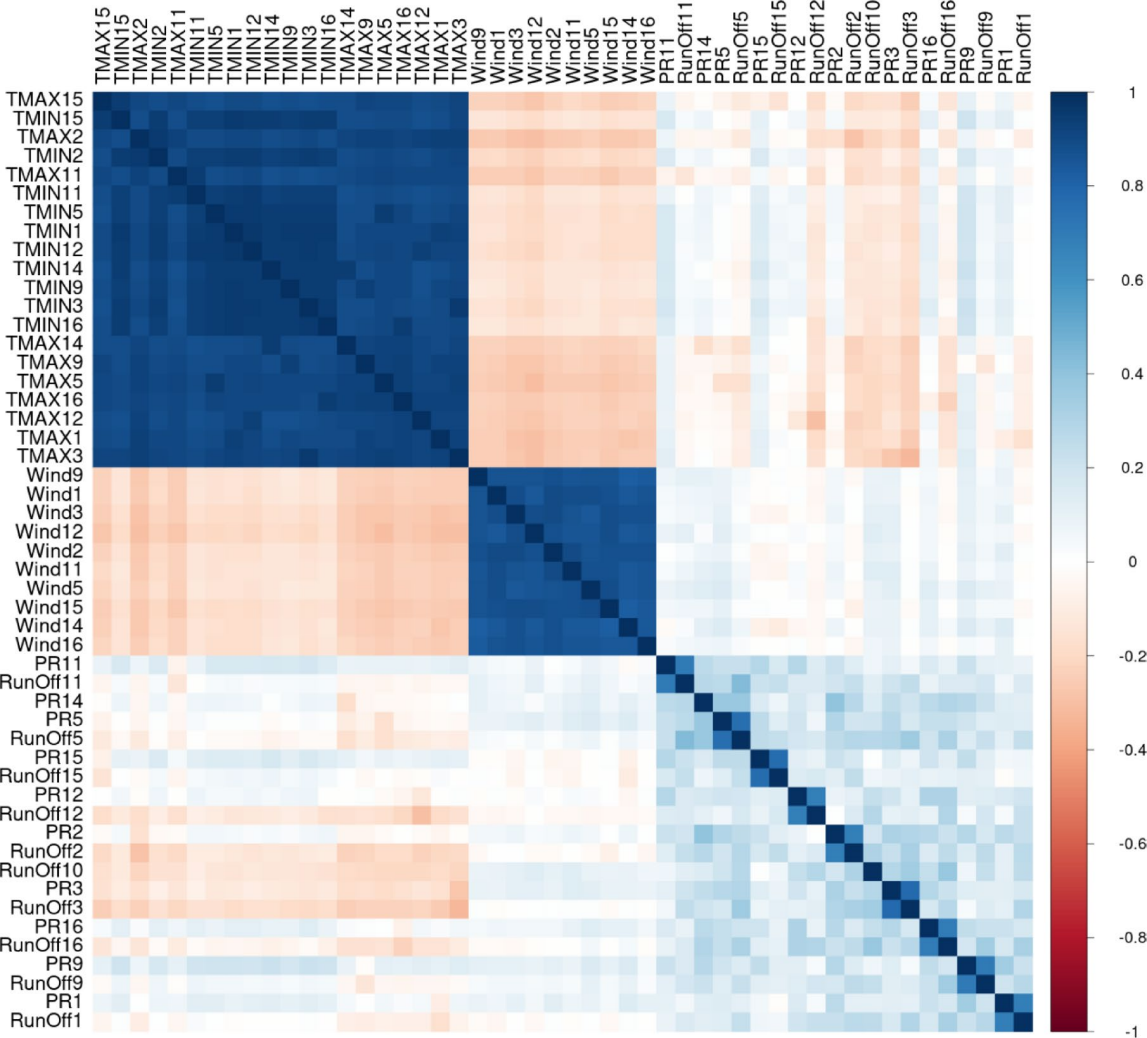
Correlation Matric for included variables in bagged DR Model for RCP 8.5.

Further inspection of all included variables in the bagged DT model for both emission scenarios was conducted. Figure 4 shows the correlation matrix for the bagged DT model for the emission scenario RCP 8.5. The correlation matrix includes the correlation value between the input pairs considered in this study. A correlation matrix quantifies the relationships between variables using correlation coefficients that range from − 1 to 1, where 1 indicates a perfect positive correlation, − 1 indicates a perfect negative correlation, and 0 indicates no correlation. In this study, the correlation matrix identifies dependencies between climate and the different variables included in the analysis. This helps determine key predictive features and enhances the model’s interpretability. It can be observed that the Temperature variables are highly correlated across different GCMs. However, it can also be observed that the temperature is inversely correlated to wind speed and runoff, but slightly correlated with precipitation. It can be concluded that the wind variables are not correlated with the precipitation and runoff, neither positively or inversely correlated, however, the precipitation and runoff variables are positively correlated for each GCM simulation, but not across different models, highlighting the need for an ensemble technique for including as many GCMs as possible to expand the range of variables in the analysis and their impact on the prediction models.

**Fig. 5:**
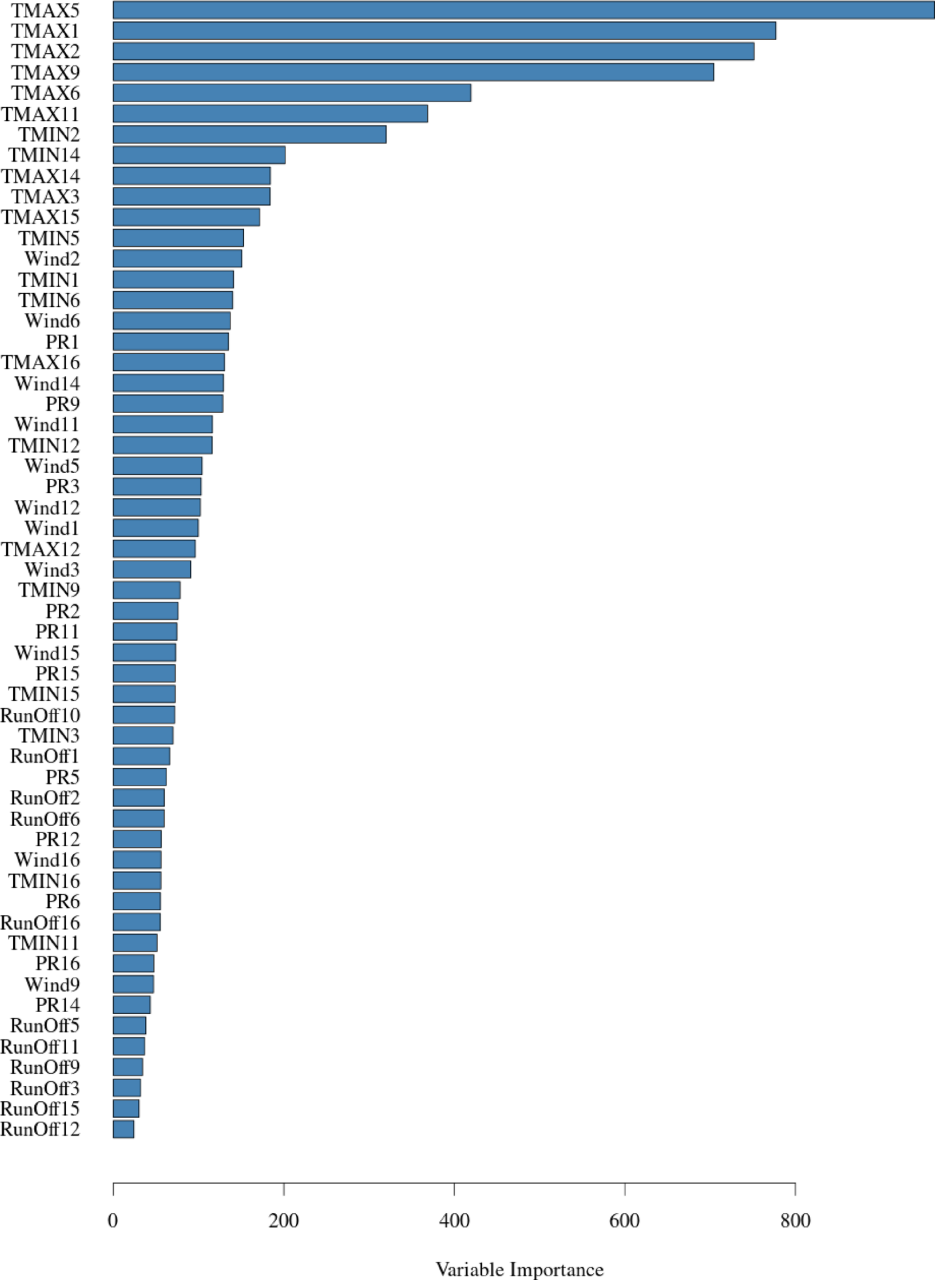
Variable importance for the included variable in bagged DT model where; (**a**) RCP 6.0, and (**b**) RCP 8.5.

The following analysis is the identifying the Variable Importance in the developed algorithm. Variable importance quantifies the contribution of each predictor to the model’s output, helping identify the most influential factors in flood resilience assessment. Higher importance values indicate stronger influence on predictions, while lower values suggest minimal impact. Figure 5 shows the variable importance (VI) bar chart for the RCP 8.5 emission scenario. We can conclude that the temperature variables are most influential in the model’s performance, having the first 15 variables based on their VI as temperature outputs of the different GCMs. It is also clear that after a certain threshold, the VI drops significantly, indicating that some GCMs have a bigger influence on the probability of correctly classifying a class, however an ensemble of multiple GCMs is needed to increase the overall total performance and include as much variability and bias elimination in the prediction algorithm.

**Fig. 6:**
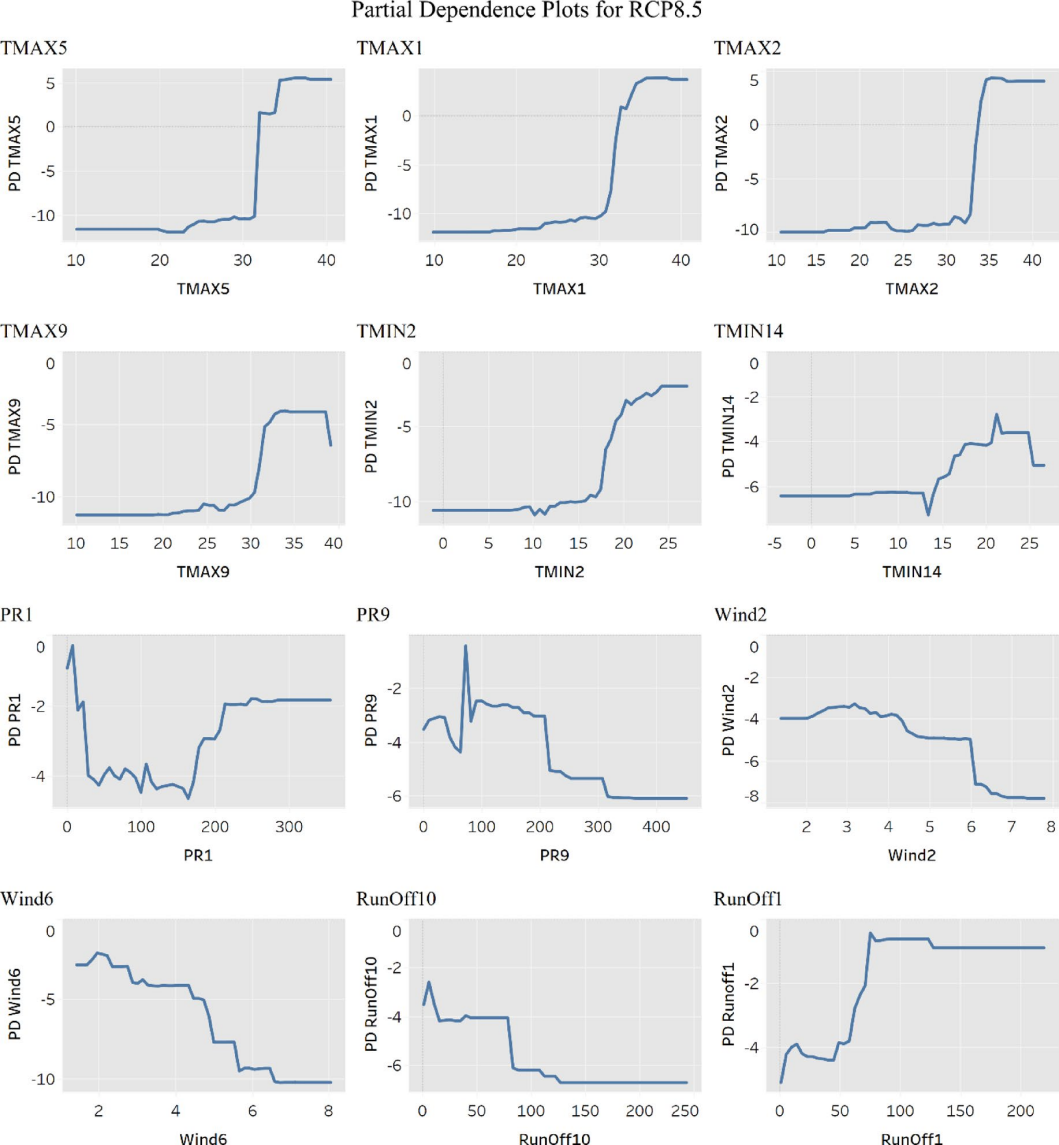
Partial dependence plots for RCP 8.5.

The last, and most essential, interpretability technique included in this study is the Partial Dependence Plots (PDP) as shown in Fig. 6. PDPs help visualize the complex interrelationships between the predicted categories and the ML model inputs from the various GCMs, where the effect of change is represented in a single-variable plot. The PDP in Fig. 6 shows the first four maximum temperature variables, the first two minimum temperatures, the first two precipitation variables, the first two wind speed variables, and the first two runoff variables based on their importance according to the VI plots as shown in Fig. 5. The values of the variables in the model are normalized and scaled for an optimum homogeneity in the ML model, to avoid skewness in the model and account for the multiple different units of measurement for all variables, it was unscaled back to its original range for the development of the PDPs, to draw interpretability insights. While it is expected that the results will vary depending on the GCM model results being inspected within the input space of the ML model and the emission scenario under investigation, the overall behavior of these variables is expected to remain the same. For the RCP 8.5 PDPs, the PDPs for the maximum temperatures all indicate a clear jump in the influence of the resilience category when the maximum temperatures are between 30 and 40 °C, showing that the risk of flooding disasters increase as the temperatures rise, the same applies for all GCMs included in the development of the predictive model. The minimum temperatures do not have an equivalent increase in predicted flood risk impact, but it shows a slight rise when monthly minimum temperature is between 15 and 30 °C. This can be attributed to the increased heat content over the Gulf of Mexico, transforming the weather into a tropical atmosphere, positively correlated to increased rainfall and a suitable climate for the development of hurricanes^33,35^. The resilience category is higher at lower runoffs, then gradually fluctuates and falls until it reaches 100 mm. However, the fluctuations observed in the PDPs for precipitation (PR1 and PR9) are from differences in the underlying climate models, as these variables originate from separate datasets with distinct corresponding temperature and runoff values. These variations influence how precipitation affects community vulnerability. Specifically, the PDP for temperature follows a consistent trend due to its direct correlation with flood patterns in the Gulf of Mexico. However, the precipitation PDP exhibits a more complex trajectory due to hydrological thresholds and infrastructure capacity. Initially, an increase in precipitation corresponds to a rise in vulnerability, as higher runoff places additional strain on drainage systems. However, once precipitation levels exceed 150 mm, the vulnerability index stabilizes, indicating that community infrastructure reaches its capacity to manage additional rainfall. Beyond 220 mm, the PDP plateaus, suggesting that further increases in precipitation do not proportionally affect vulnerability. This pattern reflects a saturation effect, where the system has reached its upper threshold, and additional rainfall no longer influences the vulnerability index.

Using such interpretability methods as shown in Figs. 4, 5 and 6 proves its employability in better understanding the behavior of the different variables within the ML algorithm, their influence on the prediction output, and the interrelationship and interdependencies between these variables. This interpretability feature ensures that the output of the ML prediction algorithm can be investigated more thoroughly, and the behavior of the input-output variables can be explained.

### Scalable geospatial framework and prediction

By running the prediction algorithm on the GCMs projections till the year 2050, this employed framework captures the change in resilience of the chosen 45 counties presented in Table 2. A Spatio-temporal analysis was conducted to visualize the effect of climate change on the built environment and identify the vulnerabilities and climate change’s impact on flood exposure and resilience.

**Fig. 7:**
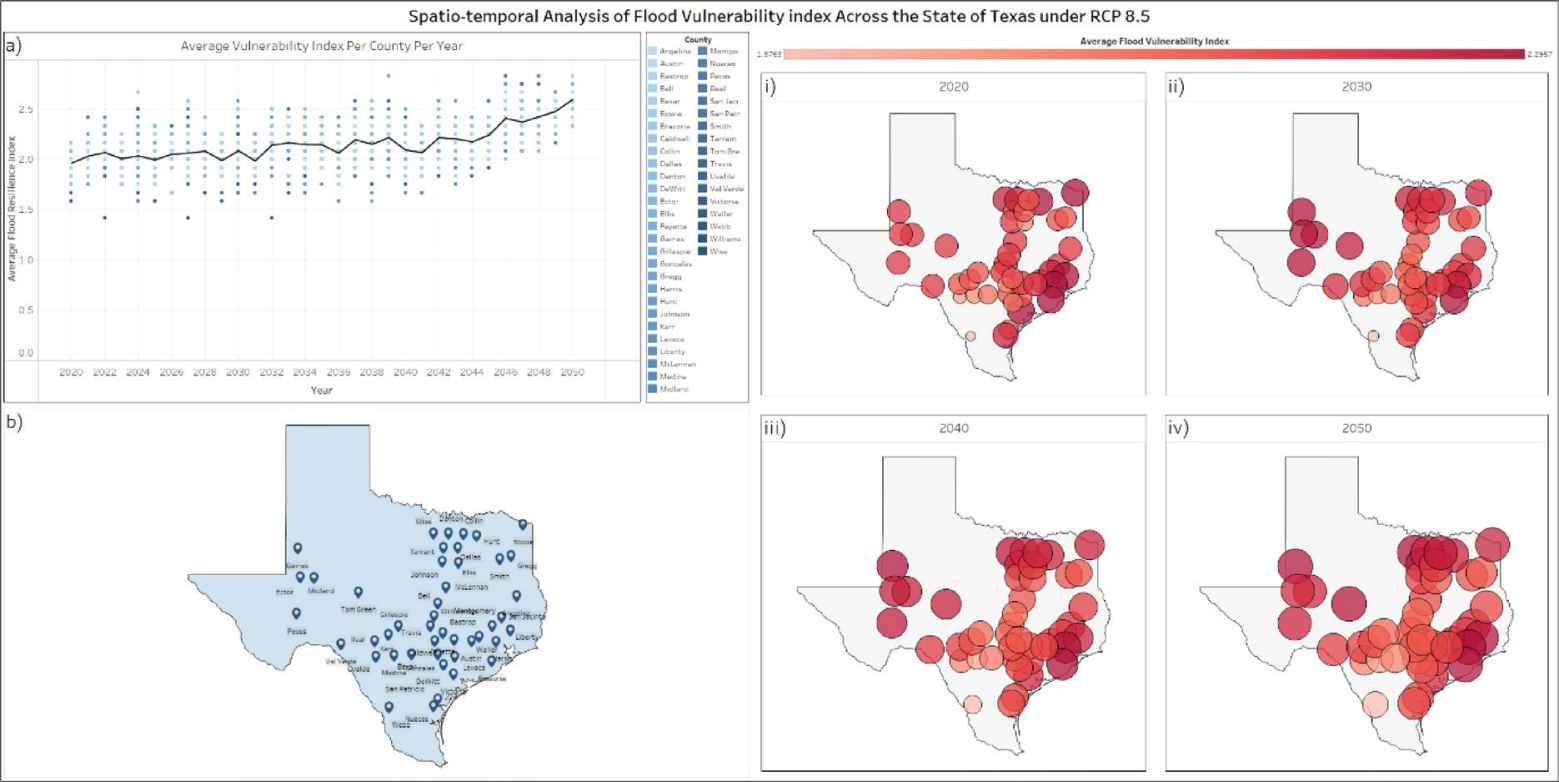
Spatio-temporal Model Output visualization for RCP 8.5, where; (**a**) yearly average per county per year, with a running average for all included counties, (**b**) spatial distribution of included counties and their GCM’s stations, (i) the spatial distribution of average Vulnerability Index per county in the year 2020, (ii) spatial distribution of average Vulnerability Index per county till the year 2030, (iii) spatial distribution of average Vulnerability Index per county till the year 2040, (iv) spatial distribution of average Vulnerability Index per county till the year 2050.

Figure 7a is a temporal distribution of the yearly average resilience category per county per year between the years 2020 and 2050, Fig. 7b is the spatial distribution of the counties involved in the analysis, Fig. 7(i) is a multi-layer spatial distribution of the year 2020 where the location of each county is identified by a circle, the size and color of the circle represents the running cumulative average resilience category, Fig. 7(ii) a multi-layer spatial distribution of the running cumulative average until the year 2030, Fig. 7(iii) a multi-layer spatial distribution of the running cumulative average until the year 2040, Fig. 7(iv) a multi-layer spatial distribution of the running cumulative average until the year 2050, also differentiated by size and color for visualization. The average category between the years 2020 and 2030 is 2.2, between the years 2030 and 2040 the average is 2.3, and 2.52 between the years 2040 and 2050. The historical recorded average damage per decade between the years 1996 and 2020 is $5,702 M, the projected damage based on the RCP 8.5 scenario between the years 2020 and 2030 is $6400 M, at an increase of 12.2% in monetary damage, between the years 2030 and 2040 the projected monetary loss is $6692 M at an increase of 17.4%, and between the years 2040 and 2050, the projected monetary loss is at $7331 M at an increase of 28%. Similarly, the resilience category is also an indicator for other socio-economic components, like injuries, fatalities, evacuations, and the downtime of the community following the flood event. The indicator shows an increase of 28% per decade in these components leading up to the year 2050. This increase doesn’t only show the need for intervention measures as mentioned earlier, but also the need for global effort to reduce carbon emissions and GHG into the atmosphere, in a desperate attempt to evade the RCP 8.5 scenario, since it is now almost inevitable to actualize an intermediate emission scenario with the current global efforts. This global effort, coupled with multiple resilience-guided flood risk analysis could potentially save billions of dollars from tax-payers money.

## Discussion

Flood risk remains one of the costliest and most disruptive natural hazards worldwide. The IPCC 2021 report states that extreme rainfall events are increasing in severity and frequency over the next decade, with an expected rise of average sea water level by 2.0 m by the year 2100. This change is also governed by the global climate policies in place, and how the world abides by them on a global scale, dictating the direction upon which GHG emissions would determine which RCP scenario the climate would follow. These findings ascertain the need for a comprehensive flood-risk prediction methodology that is resilience-centric, employable in multiple scenarios and across a wide range of urban, geographical, and climatological properties.

The work presented herein develops a scalable framework and presents a comprehensive methodology for incorporating climate change impact with numerous community resilience features. This work aims at: (i) identifying variables that comprehensively represent the resilience goals for incorporation within a data-driven multi-stage model, (ii) employ the developed dataset to produce resilience indices appropriately representing the features of the community under investigation, ranging from the quality of the complex infrastructure system forming the functionality of the society, to the expected damages and the impact on the livelihood of the inhabitants, (iii) couple the employed indices with climate change scenarios to develop a prediction model to investigate the impact of climate change on future resilience trajectory of the built environment, and (iv) employ the developed models into developing a spatio-temporal analysis of the area under investigation, identifying future trends in community resilience, and the potential vulnerabilities in the built environment.

In this study, the BCSD CMIP5 models were employed for climate modeling under multiple emission scenarios, with 16 Global Climate Models employed. The Study also employs the disaster data records developed by the National Weather Service (NWS) from the years 1996–2020. Spatial analysis was conducted using the employed resilience categories, identifying the State of Texas as the one befalling the most monetary damage, and the most recorded flood disasters. Henceforth, 45 test locations (i.e., counties) were identified within the state of Texas for the CMIP5 GCMs simulations for the climate modelling, and the resulting models were coupled with the Vulnerability Index on each of the recorded disaster dataset observation.

Multiple Interpretability techniques were employed to interpret the results of the ML model to transform the model from its Black-box nature to a more readable model, enabling decision and policy makers to draw reliable managerial insights and information for the development of the much-needed mitigation plans. The interpretability methods employed in this study identified the following insights: (1) the behavior and relative influence of the features across the multiple ensemble GCMs employed in the development of the algorithm is similar, identifying that some assumptions in the simulation of the different GCMs do not impact the behavior of the features, but rather varies in term of accuracy and prediction trajectory it provides, (2) the maximum and minimum temperatures are the most influential climate information in all models, correlating (whether directly or inversely) with most of the included features (i.e., precipitation, runoff, and windspeed), (3) the impact of the temperature on the community resilience increases exponentially between 30 and 40 °C for average maximum daily temperatures, and 15–30 °C for average minimum temperature in the region where the tests were conducted, (4) the influence of windspeed on the resilience categories is increasing up to 6 m/s, then starts slowing down significantly, and (5) the influence of the total precipitation comes to a halt beyond the 200 mm threshold, indicating a maximum damage reached in the case of extreme event, or that the infrastructure (i.e., drainage network) running at full capacity. Notwithstanding the interpretability of the ML model, the prediction results also provided key insights into the inherent resilience of the built environment. Showing an expected 28% increase in expected damage (monetary and otherwise) till the year 2050, and $1.8B in damage per decade for RCP 8.5 scenario. However, this damage is only in the 45 counties included in this study, with a much larger expected increase in flood damage for the entire state of Texas.

In conclusion, this study presents a scalable geospatial framework designed to enhance climate resilience planning by integrating spatial, environmental, and socio-economic datasets. By leveraging multi-scale geospatial analytics, the framework provides a dynamic decision-support tool capable of assessing vulnerabilities, modeling climate-induced risks, and supporting evidence-based policy interventions. Through the application of this framework in Texas, the study demonstrates how our proposed framework can inform large-scale resilience assessments by integrating diverse data sources into a unified predictive model. The results illustrate that multi-scale geospatial analytics can significantly enhance climate risk modeling, allowing policymakers to better understand exposure, adaptive capacity, and long-term vulnerability trends.

While this research establishes a methodological foundation for climate risk and adaptation planning, it also highlights key areas for future development. To enhance predictive capabilities, future work should explore the integration of real-time data streams, such as live weather feeds, such as real-time precipitation, streamflow, and soil moisture data, the framework could be continuously updated to reflect the most current hazard conditions, enhancing its practical applicability for decision-makers. Additionally, incorporating physics-based hazard modeling alongside machine learning techniques could refine the framework’s ability to simulate climate-related disruptions and their cascading effects.

While our framework explores a wide ensemble of climate projections through multiple GCM subsets, the results presented in this study should be interpreted as indicative trends rather than precise predictions. Given the inherent uncertainty in climate modeling, particularly across GCMs, emission scenarios, and impact pathways, we acknowledge that the 28% projected increase in damages under RCP 8.5 by 2050 is a deterministic estimate and not a probabilistic forecast. The current version of the framework does not incorporate confidence intervals or probabilistic spread but is fully adaptable to include uncertainty quantification. Future work will build upon this by incorporating formal representations of uncertainty to improve interpretability and decision-making utility.

It is important to clarify that the framework presented herein is not a fully realized Digital Twin but a scalable geospatial architecture that can evolve toward one. Unlike conventional digital twin systems, which continuously assimilate real time sensor data and operate at high spatial and temporal resolutions, the present study demonstrates the feasibility of a data driven, modular framework that can be extended to incorporate these features in future applications. This distinction positions the framework as an intermediate, resource efficient pathway bridging conventional static models and dynamic digital twin environments.

Ultimately, the framework represents a significant advancement in climate resilience planning, offering a scalable, adaptive, and data-driven methodology for decision-makers to proactively manage and mitigate climate risks. By integrating geospatial intelligence with climate analytics, this framework lays the groundwork for a next-generation climate adaptation tool that can support infrastructure resilience, disaster preparedness, and sustainable urban planning.

## Future recommendation

The methodology adopted herein can be further developed into a global prediction algorithm, acting as an early warning system, and a cornerstone for a comprehensive management system for the built environment—a *Comprehensive City Twin*. However, to achieve said potential, numerous requirements need to be met (e.g., availability of comprehensive enough datasets with enough variability for the development of the categories, and availability of the stations collecting climate information and observations for the development of the climate modeling and projections). The analysis also calls for immediate global intervention to steer the global trajectory to a less severe emission scenario, since the expected impact on the built environment increases two-fold from one scenario to another in the next 30 years. Future research should further explore hybrid methodologies that combine data-driven insights with process-based simulations, reinforcing the framework’s ability to guide climate adaptation strategies in increasingly uncertain environments. Additionally, cloud-based integration should also be explored, enabling a scalable and automated system for real-time disaster prediction. Expanding the range of dependent and independent variables to include additional disaster types and systemic climate risks could further enhance the framework’s applicability across different regions and hazard contexts.

Finally, Increasing the temporal resolution from a monthly to a daily scale could further improve predictive capabilities by enabling more frequent flood risk assessments. This could support the development of early-warning systems that provide municipalities with timely alerts for proactive preparedness measures. However, implementing higher-resolution data requires additional datasets, such as daily climate records, real-time flood gauge measurements, and high-frequency hydrological observations, to refine vulnerability assessments. From a machine learning perspective, incorporating higher-resolution data introduces computational and methodological challenges. These include optimizing feature selection processes, refining hyperparameters, and addressing data sparsity or inconsistencies that occur at finer temporal scales.

## Methods

### Datasets

#### Developing the resilience and vulnerability based categories

For part (a) adopted in this study, the historical disaster data records from the National Weather service (NWS) were adopted. This dataset is used for the derivation of the resilience-based categories employed in this study. The NWS is considered one of the longest-run organizations concerned with recording annual flood damage in the United States^37^. The dataset employed here while compiled into the database of the NWS, was nonetheless gathered by third-party organizations and data-collection agencies. Although these agencies followed the standards and guidelines provided by the NWS, the diversity, quality and quantity of the collected data are highly dependent on the resources of these organizations, and their constraints, financial and otherwise^28^. This dataset of historical disasters contains a total of 49,775 records between 1996 and 2020 for the United States mainland. For each data record, there are multiple variables—start time, end time, geographical location, year, month, duration, and related damages. The recorded damages in this dataset include the direct and indirect injuries and fatalities, crop damage and property damages. The start and end data of the flood event were used to calculate the total event duration, representing a portion of the down time (i.e., Rapidity), and the month was used to represent the seasonality of the flood event. To estimate projected economic damages from categorical resilience predictions, we employed a post-processing step that maps predicted resilience classes to monetary loss estimates. This mapping was based on historical FEMA and NOAA datasets that report inflation-adjusted damages at the county level. Damages were normalized to the date of analysis’s USD using the Consumer Price Index (CPI). For each class, average historical damage figures were computed and used as multipliers to extrapolate losses under future scenarios. The underlying assumption is that regions predicted to fall into a higher vulnerability class would incur higher damages in line with their historical analogs. The approach includes back-testing against events to validate the scale of predicted losses^28,38–40^. For reliable resilience-guided insights to be drawn from this analysis, the features of the flood hazard need to be considered, this information is implicitly stated through the typical spatial and temporal attributes of the hazards in the dataset, where each community is defined by its inherent characteristics. It is essential to identify that the damages (monetary and otherwise) recorded in this dataset are all direct damages, resulting from the direct contact of the flooding water with the structures or the components of the community, and short-term indirect losses (i.e., losses due to displacements, and immediate mitigation measures, not accounting for the opportunity loss or the indirect damages of resulting from this event). To that end, the term “Flood Event” mentioned in this dataset refers to the flooding component of any multi-hazard event; such that if a flood is a result of a cyclone or a tornado, the recorded damages in this dataset only relate to the flooding component of this multi-hazard environment^37,39,41^.

#### Climate information corresponding to historical disaster records

This data was extracted from the Global Historical Climatology Network (GHCN-Daily) of the National Center for Environmental Information^42^. For a comprehensive analysis with reliable information and insights, a diverse and comprehensive dataset that includes all the relative variables with enough observations over the years to avoid bias needs to be utilized. To that end, the purpose of this study is to develop a methodology that can employ global climate models to predict future changes in the inherent resilience of the built environment. Using historical data, as proposed in the study by Abdel-Mooty et al.^43^ although beneficial and critical to the accurate synchronization of the dataset, will not aid in the development of an accurate prediction algorithm.

#### Climate change projections

The Bias Correction with Spatial Disaggregation (BCSD) Downscaled Coupled Model Intercomparison Project—Phase 5 (CMIP5) is employed in this study as the most complete and tested GCMs^44,45^. This dataset employs a large ensemble of GCMs based on multiple Green House Gas (GHG) emission scenarios. The gridding of the dataset is accordingly adjusted to a course 1^o^ resolution, with a downscaled spatial resolution of 1/8^o^. The bias identification was based on the overlapping period in the historical observations and the modeled GCMs from the years 1950–1999 and was carried on a temporal and spatial basis (i.e., for common months and basins) with the location provided at a grid-cell resolution. The correction of the bias was made by looking at the associated rank probability (p) at a certain timestep from the GCMs historical quantile map and comparing it to that of the historical dataset. This results in all the bias-corrected GCM results being associated with monthly Cumulative Distribution Functions (CDF). The results are then linked and used in correcting the bias of the future projections from those GCMs in the CMIP5 dataset^44–46^.

This study further incorporates outputs from both Global Climate Models (GCMs) and Earth System Models (ESMs) provided in the CMIP5 archive. These models simulate key climate processes and deliver projections for essential variables including precipitation, near-surface temperature, and surface runoff. The runoff variable (commonly referenced as “mrro”) is derived from the land-surface hydrology modules embedded within ESMs, capturing dynamic interactions between atmospheric forcing and terrestrial water balance components. This multi-model ensemble approach enhances the robustness of climate inputs used in the resilience evaluation presented herein^47,48^.

While this framework leverages indirect damages as a proxy for rapidity, defined here as the speed of community recovery following an event, we recognize that this introduces assumptions about the relationship between disruption, recovery time, and measurable damages. These assumptions may not universally hold across geographies or future climate conditions due to differences in socio-economic systems, infrastructure robustness, and policy responses. Since these proxies are embedded within the dependent variable used for model training, they may influence the generalizability of the predictions when applied beyond the historical record. Nonetheless, this proxy-based approach offers a practical solution in data-sparse environments and can serve as a foundation for refinement as more direct or high-resolution resilience indicators (e.g., real-time recovery data, infrastructure restoration timelines) become available. We therefore view this model as an adaptable first step, with clear limitations that should be acknowledged when interpreting projections.

Climate model bias correction is commonly employed to adjust systematic deviations in climate projections, improving their agreement with historical observations. While this process enhances model usability, recent studies highlight several limitations, particularly in its representation of extreme events, inter-variable dependencies, and long-term climate signals^49,50^. Given that the present framework integrates climate model outputs, it is essential to acknowledge these uncertainties and their implications for resilience assessments^51^.

One major concern is the misrepresentation of extreme climate events. Bias correction techniques often assume that model biases remain constant over time, yet under non-stationary climate conditions, such assumptions may not hold. This can result in the underestimation or overestimation of rare events, particularly those that fall outside the range of historical observations (Frontiers in Environmental Science). Additionally, some bias correction approaches alter the internal variability of models, potentially distorting key climate signals relevant to resilience assessments^50,52^. Given these limitations, the framework presented herein does not assume bias-corrected data to be inherently superior and instead acknowledges its potential distortions in resilience modeling.

The Intergovernmental Panel on Climate Change (IPCC) identified multiple benchmark GHG emission scenarios as the Representative Concentration Pathway (RCPs) (i.e., RCP 2.6, RCP 4.5, RCP 6.0, and RCP 8.5)^53^. Current consensus states that GHG emissions have crossed the RCP 2.6 scenario and nearing that of RCP 4.5^54,55^. While RCP 4.5 and RCP 6.0 are considered “Intermediate Scenarios”, the distinction between both scenarios is GHG emissions are beyond the year 2050, which makes the distinction between them minimal for analyses that extend to the year 2050. It is recommended by the IPCC and other agencies that analysis for climate projections would include an intermediate and a severe scenario, with both RCP 4.5 and RCP 6.0 being comparable, the authors elected to deploy RCP 6.0^54^ (along with RCP 8.5, and included in the supplementary information with this manuscript) to highlight the need for global intervention, and to showcase the applicability of the proposed framework, and highlighting its generalizability to accommodate other emission scenarios. The RCPs are concentration-driven pathways defined by the radiative forcing expected by 2100. In contrast, the newer Shared Socioeconomic Pathways (SSPs), used in updated models CMIP6, incorporate information about global socio-economic development in addition to emissions trajectories. SSPs provide a more comprehensive view of future scenarios by incorporating variables such as population growth, economic development, and technological advancements^56^. While the SSP-based CMIP6 framework is more comprehensive, CMIP5 was used in this study due to its maturity and the wide availability of downscaled, bias-corrected datasets at the time of conducting this research. Specifically, the Bias Correction with Spatial Disaggregation (BCSD) dataset offered a robust and validated foundation for spatially resolved hydrological variable projections across multiple GCMs^56^. Therefore, the results presented here should be interpreted within the CMIP5 context and not as generalizable to CMIP6. That said, the proposed framework is model-agnostic and remains adaptable to incorporate CMIP6 or subsequent climate model generations as those datasets become more accessible and standardized.

The dataset used in this study provides climate variables such as maximum and minimum surface air temperature (°C), wind speed (m/s), total runoff (mm), and precipitation (mm). These variables were extracted from 16 GCMs under the RCP 8.5 scenario, as summarized in Table 3. The use of CMIP5 in this context ensures consistency and replicability across all selected scenarios, while the methodology itself remains fully adaptable to CMIP6 or future modeling efforts as more datasets become available.

**Table 3:** The 16 global climate models for RCP 8.5.

	Model name	Institution	Modeling center
1	ACCESS1.0	CSIRO (Commonwealth Scientific and Industrial Research Organization, Australia), and BOM (Bureau of Meteorology, Australia)	CSIRO-BOM
2	BCC-CSM1.1	Beijing Climate Center, China Meteorological Administration	BCC
3	BCC-CSM1.1(m)		
4	CanESM2	Canadian Centre for Climate Modelling and Analysis	CCCma
5	CCSM 4	National Center for Atmospheric Research	NCAR
6	CESM1(BGC)	National Science Foundation, Department of Energy, National Center for Atmospheric Research	NSF-DOE-NCAR
7	CESM1(CAM5)		
8	CMCC-CM	Centro Euro-Mediterraneo per I Cambiamenti Climatici	CMCC
9	CNRM-CM5	Centre National de Recherches Meteorologiques/Centre Europeen de Recherche et Formation Avancees en Calcul Scientifique	CNRM-CERFACS
10	CSIRO-MK3.6.0	Commonwealth Scientific and Industrial Research Organization in collaboration with the Queensland Climate Change Centre of Excellence	CSIRO-QCCCE
11	FGOALS-g2	LASG, Institute of Atmospheric Physics, Chinese Academy of Sciences; and CESS, Tsinghua University	LASG-CESS
12	FIO-ESM	The First Institute of Oceanography, SOA, China	FIO
13	GFDL-CM3	Geophysical Fluid Dynamics Laboratory	NOAA-GFDL
14	GFDL-ESM2G		
15	GFDL-ESM2M		
16	GISS-E2-R	NASA Goddard Institute for Space Studies	NASA GISS

For this stage in the methodology, the historical data from the GCMs were compared to the recorded data, and the models with the least bias were selected to create an ensemble for the ML prediction algorithm.

### Machine learning model architectures

#### ML model selection and interpretation

ML models have been gaining increased traction in the field of community resilience and anthropic and natural hazards^26,43,57–60^. For the study presented herein, supervised ML models were employed to predict the future trajectory of community flood resilience categories using the hydrological information resulting from the employed GCMs. This multiclass classification heavily depends on the variables included in the development of the algorithm^61^. As such, different techniques were deployed on this dataset to develop the most accurate interpretable results for the development of reliable managerial insights for decision makers and policy developers.

ML models are typically referred to as “Black Box” models, while this definition is accurate in most cases. However, some ML algorithms are termed “Glass Box” algorithm (e.g., Decision Trees (DT), Random Forest (RF)) by introducing means of interpretability techniques and rules that can be set to enhance the interrelation between the model output and input variables, allowing the users to draw the required insights^62–64^. Random Forests is an ensemble technique, adopting multiple DTs by aggregating their results and likelihood predictions, which improves the performance metrics of the model, but imposes a challenge for interpretability^65^. This study adopts these ML algorithms to develop an empirical framework for resilience prediction (Fig. 1) for identification of vulnerabilities in the trajectory of the current built environment. With the need to employ data preprocessing, identifying the interdependencies within a dataset, and addressing missing results through data cleaning and imputation, ensuring the reliability of the data and eliminating the skewness and any induced biases^66,67^. In the study herein, the independent variable is class-based, as such, multiclass classification techniques are adopted. Additionally, for performance enhancement, ensemble techniques will be employed to complement the adopted algorithm (i.e., bagging, random forest, boosting)^68–70^.

Within the supervised ML techniques, there are the Classification and Regression Trees (CART) algorithms, where the classification trees are most suitable for the prediction of categorical (discriminate) independent variables, as opposed to regression trees that are more suitable to continuous variables^25^. DT are based on a binary recursive partitioning algorithm with a set of rules (i.e., partitioning steps) that depend on their preceding steps^25,71^. In the classification tree algorithm, this feature selection process is based on information gain (i.e., decrease in entropy) or Gini index^71,72^. Where Entropy (*E*) is the measure of purity of the sample, the Information gain (*g*) is the decrease in Entropy (*E*) after a split based on a feature or attribute (*A*) in the training dataset (*T*), and is expressed in [Eq. (1)] through [Eq. (3)]^73^:

1 $$g\left(T,A\right)=E\left(T\right)-E\left(T|A\right)$$

Such that:

2 $$E\left(T\right)=-\sum _{k=1}^{k}\frac{\left|{C}_{k}\right|}{\left|T\right|}{\mathrm{log}}_{2}\frac{\left|{C}_{k}\right|}{\left|T\right|}$$

3 $$E\left(T|A\right)=\sum_{j=1}^{\left|A\right|}\frac{\left|{T}_{j}\right|}{\left|T\right|}\sum_{k=1}^{K}\frac{\left|{T}_{jk}\right|}{\left|{T}_{j}\right|}{\mathrm{log}}_{2}\frac{\left|{T}_{jk}\right|}{\left|{T}_{j}\right|} = \sum _{j=1}^{\left|A\right|}\frac{\left|{T}_{j}\right|}{\left|T\right|}E\left({T}_{j}\right|A)$$

Where *k* is the number of class (with a total of *K* classes); C_k_ is the sample assigned to class *k*,* T*_j_ is the sample in the *T* dataset corresponding to *j*^th^ value of attribute *A*; *T*_jk_ is the *j*^th^ sample in attribute *A* that is assigned to class *K*; and |*A*| is the number of values of attribute *A*. Higher values of the Gini Index and higher information gain (*g*) denotes to a more important feature within the CART classification algorithm^71^. However, to enhance the performance of the model, numerous ensemble techniques were adopted in this study, including bagging, boosting, and random forest^69,70^.

*Bagging* is a form of bootstrapping technique, which is a random sampling process of the data, taken by replacement, where each datapoint can be available for selection in multiple subsequent models, while still using all the predictors in the sampling process^74^. In bagging however, the aggregating technique is used for fitting multiple versions of the model within the training dataset, where each model is then used in the training of the DT model, followed by averaging all the predictions, providing a more reliable and robust model than a single DT^69,72,75^. On the other hand, *Random Forest* (RF) models are considered a step further and an extension to Bagging techniques for model performance enhancement. In RF, the predictors are also randomized at each node at a split within the DT rather than doing so iteratively. Subsequently, the results are the aggregation of the prediction from the entire set of trees^69,76–79^.

### Model performance measures

The models employed for the analysis herein are: (i) Decision trees with 100-fold cross validation, (ii) Bagged decision trees, tested with up to 15,000 bootstrap replications as an ensemble method, with a minimum split of 4, (iii) RF models with the Out-of-Bag Error tested for up to 3000 trees, showing a uniform plateau after 500 trees, indicating the unnecessity of increasing the number of trees in the forest more than 500, with four randomly sampled variables at each split, and a shrinkage parameter of 0.01^76^. The overall model misclassification error was utilized to identify the highest performing models (Eq. 4). However, with the skewness of the data, the multiclass nature of the independent variable, and for further interpretability for the models, more in-depth measures needed to be utilized. As such, the Precision, Sensitivity (i.e., Recall), and F1-score for each category were utilized in both datasets, namely training and testing. *Precision* is the measure of accuracy of each class in the prediction algorithm, denoted by the number of accurately predicted datapoints in that class as shown in (Eq. 5). Recall on the other hand is the ratio between the accurate predictions to all correct examples in the dataset, it represents the correctness of the classification results of the ML model (Eq. (6)). Finally, the F1-score, is the integration of both the Precision and Recall for the classification algorithm, where the concerns of both measures is balanced out (Eq. 7)^76,80^.

4 $$Total Misclassification= \frac{\sum _{r=1}^{K}\sum _{c=1}^{K}{N}_{rc}-\sum _{i=1}^{K}{TP}_{i}}{\sum _{r=1}^{K}\sum _{c=1}^{K}{N}_{rc}}$$

5 $$Precision= \frac{TP}{TP+FP}$$

6 $$Recall= \frac{TP}{TP+FN}$$

7 $$F1\--score= 2*\frac{Precision*Recall}{Precision+Recall}$$

Where K = number of Classes in the independent variable; N is the count number of observations allocated in each cell of the confusion matrix; TP = True Positive, which is the number of accurately predicted observations in a given class; FP = False Positive is the count number of predictions incorrectly assigned to a category; FN = False Negative is the count number of observations incorrectly assigned to a wrong class^81^.

### Model interpretability techniques

The ambiguity associated with these ML algorithms hindered the progressive utilization of such models in fields such as structural and civil engineering and community resilience planning^82,83^. While the model employed herein is a post hoc model, means it presents an explanation to the ML model following the model’s employment and obtaining the results, post hoc interpretability models’ accuracy depends on the employed dataset’s structure, and is domain specific. For the study presented herein, the employed interpretability techniques provide high accuracy and adequate explanation of the results of the model. However, these methods should be used with care, and avoided when possible, with high-stake decisions, and explore ad hoc models instead^84^. This phase of the methodology employs Partial Dependence Plots (PDP) as a tool for interpretability, where the input-output relationship between most variables is explored and depicted into complex-linear relationships^82^. Partial Dependence Plots (PDPs) are a model-agnostic interpretability technique used to visualize the marginal effect of one or more predictor variables on a model’s predictions. PDPs compute the expected prediction by averaging over all possible values of the remaining features, thereby isolating the influence of the selected variable(s). This approach provides insights into whether a feature has a linear, nonlinear, or threshold effect on the outcome. However, PDPs assume feature independence, which can lead to misleading interpretations when strong correlations exist between predictors. Despite this limitation, PDPs remain a valuable tool for understanding global feature contributions. In PDPs, the impact of the input features— whether single input or group of variables (e.g., Temperature or Temperature and Precipitation) is explored as the average prediction corresponding to the range of values for the other unused input features^85,86^. Another limitation of PDPs is that sometimes the heterogenous effect can be hidden, which is addressed by the normalization and scaling of the different variables in the interpretability techniques. Despite this limitation, PDPs remain a valuable tool for understanding global feature contributions. Partial Dependence Plots (PDPs) reveal how a feature influences model prediction. It is important to note that the observed thresholds presented in the Partial Dependence Plots (PDPs) should not be interpreted as definitive boundaries. PDPs are sensitive to correlations between predictors, and as demonstrated in our correlation matrix (Fig. 4), several climate variables exhibit significant dependencies. Therefore, the insights derived from PDPs are heuristic in nature and should be understood as indicative model responses rather than universal or mechanistic relationships. A flat line indicates no effect, while an upward or downward slope suggests a positive or negative correlation, respectively. Nonlinear patterns indicate complex relationships, potentially involving interactions or threshold effects. These insights help assess feature importance and model behavior. Another method for interpretability employed in this study is the Variable Importance (VI) algorithm. VI is used to infer the influence of a variable (e.g., Maximum Temperature of Model ACCESS1.0) on the prediction process, and the output of the model. This method quantifies the extent upon which the model relies on each of the involved feature, identifying the most influential variables within the dataset for further investigation. The VI included in this analysis is based on the Receiver Operating Characteristics (ROC) curve analysis conducted on each predictor. The ROC demonstrates the model’s susceptibility to incorrectly classify the observations in the dataset, where a series of cutoff methods are applied to the predictors for the prediction to take place. The area of the ROC curve is then calculated for each class pair (i.e., Category 1 vs. Category2, Category 2 vs. Category 3, etc.) using the trapezoidal rule, then the maximum area under the curve across the relevant pair-wise curves is considered the VI in the model^87^. This is also done by evaluating the corresponding increase in model performance measure (e.g., information gain)^82,83,85,86,88^.

### Non-stationarities in machine learning model

To mitigate the risk of unreliable extrapolations when climate variables exceed historical training data, we suggest incorporating scenario-based stress testing, physics-informed machine learning (PIML), and Bayesian inference techniques. Scenario-based stress testing evaluates model robustness under extreme and unprecedented conditions, ensuring adaptability beyond observed climate patterns. Additionally, physics-informed ML methods are also proposed to integrate physical constraints into the predictive modeling process, improving generalization by enforcing domain-relevant principles. These methods embed physical constraints, conservation physical laws, and domain knowledge into the learning process, allowing the predictive component of the framework to better simulate climate hazards even under non-stationary conditions. This hybrid modeling approach leverages the predictive power of data-driven methods while ensuring consistency with known physical principles, thereby reducing the risk of spurious extrapolations. Finally, Bayesian inference enables probabilistic uncertainty quantification, allowing the framework to provide confidence intervals and risk assessments that account for climate variability. By assigning probability distributions to model parameters, this method accounts for uncertainty in climate projections and the inherent variability in resilience assessments, providing decision-makers with confidence intervals for informed decision-making. These refinements enhance the framework’s ability to support climate resilience planning under evolving environmental conditions^89,90^.

## Supplementary Information

Below is the link to the electronic supplementary material.

Supplementary Material 1

## Data Availability

The datasets used in this article are publicly available. The meteorological disaster database used in the generation of the resilience categories is provided by the NWS, a sub-agency under the National Oceanic and Atmospheric Administration (NOAA), available at ( [https://www.ncei.noaa.gov/pub/data/swdi/stormevents/csvfiles/]). The historical climate data used is provided by Global Historical Climatology Network, a sub-agency under NOAA, and is available at ( [https://www.ncdc.noaa.gov/cdo-web/search? datasetid=GHCND] ), and the BCSD CMIP5 projections and simulations can be conducted and accessed through ( [https://gdo-dcp.ucllnl.org/downscaled\_cmip\_projections/] ). All models, or codes, that support the findings of this study are available from the corresponding author and electronically at: [https://doi.org/10.5281/zenodo.7720905]
