# Supplementary material for: A scalable data driven geospatial framework for climate risk assessment
Source: Sci Rep. 2025 Dec 20;16:2495. doi: 10.1038/s41598-025-32370-7 (PMC12820248; doi:10.1038/s41598-025-32370-7)
Supplement: Supplementary file 1 — Supplementary Material 1 [file 41598_2025_32370_MOESM1_ESM.pdf]

Supplementary Information: A Scalable Data Driven Geospatial Framework for Climate Risk Assessment

Moustafa Naiem Abdel-Mooty*, Paulin Coulibaly, and Wael El-Dakhakhni
Department of Civil Engineering, McMaster University, Hamilton, Canada

Email: abdelmom@mcmaster.ca

Supplementary Note 1: Determine Optimum Model for RCP 6.0 Emission Scenario.

16 GCMs for RCP 6.0 were adopted in each of the emission scenarios, as shown in Table 1 for RCP 6.0, and Table 2 for RCP 8.5.

Supplementary Table 1: The 16 Global Climate Models for RCP 6.0

	Model Name	Institution	Modeling Center
1	BCC-CSM 1.1	Beijing Climate Center, China Meteorological Administration	BCC
2	CCSM 4	National Center for Atmospheric Research	NCAR
3	CESM1 (CAM5)	National Science Foundation, Department of Energy, National Center for Atmospheric Research	NSF-DOE-NCAR
4	CSIRO-MK3.6.0	Commonwealth Scientific and Industrial Research Organization in collaboration with the Queensland Climate Change Centre of Excellence	CSIRO-QCCCE
5	FIO-ESM	The First Institute of Oceanography, SOA, China	FIO
6	GFDL-CM3	Geophysical Fluid Dynamics Laboratory	NOAA-GFDL
7	GFDL-ESM2G		
8	GFDL-ESM2M		
9	GISS-E2-R	NASA Goddard Institute for Space Studies	NASA GISS
10	HadGEM2-AO	National Institute of Meteorological Research/Korea Meteorological Administration	NIMR/KMA
11	HadGEM2-ES	Met Office Hadley Centre (additional HadGEM2-ES realizations contributed by Instituto Nacional de Pesquisas Espaciais)	MOHC (additional realizations by INPE)
12	IPSL-CM5A-MR	Institute Pierre-Simon Laplace	IPSL
13	MIROC-ESM	Japan Agency for Marine-Earth Science and Technology, Atmosphere and Ocean Research Institute (The University of Tokyo), and National Institute for Environmental Studies	MIROC
14	MIROC-ESM-CHEM		
15	MIROC5		
16	NorESM1-M	Norwegian Climate Centre	NCC

The Brute-Force method is used to scan through all possible combinations from the included 16 Global Climate Models (GCM). Supplementary Figure 1 shows the results of all possible

combinations for GHG emission scenario RCP 6.0, identifying the model number 32,517 as the one with least Misclassification error (3.05%) at only 8 GCMs included in the analysis (GCMs: 1, 6, 7, 9, 10, 11, 15, and 16, with their details in Supplementary Table 1).

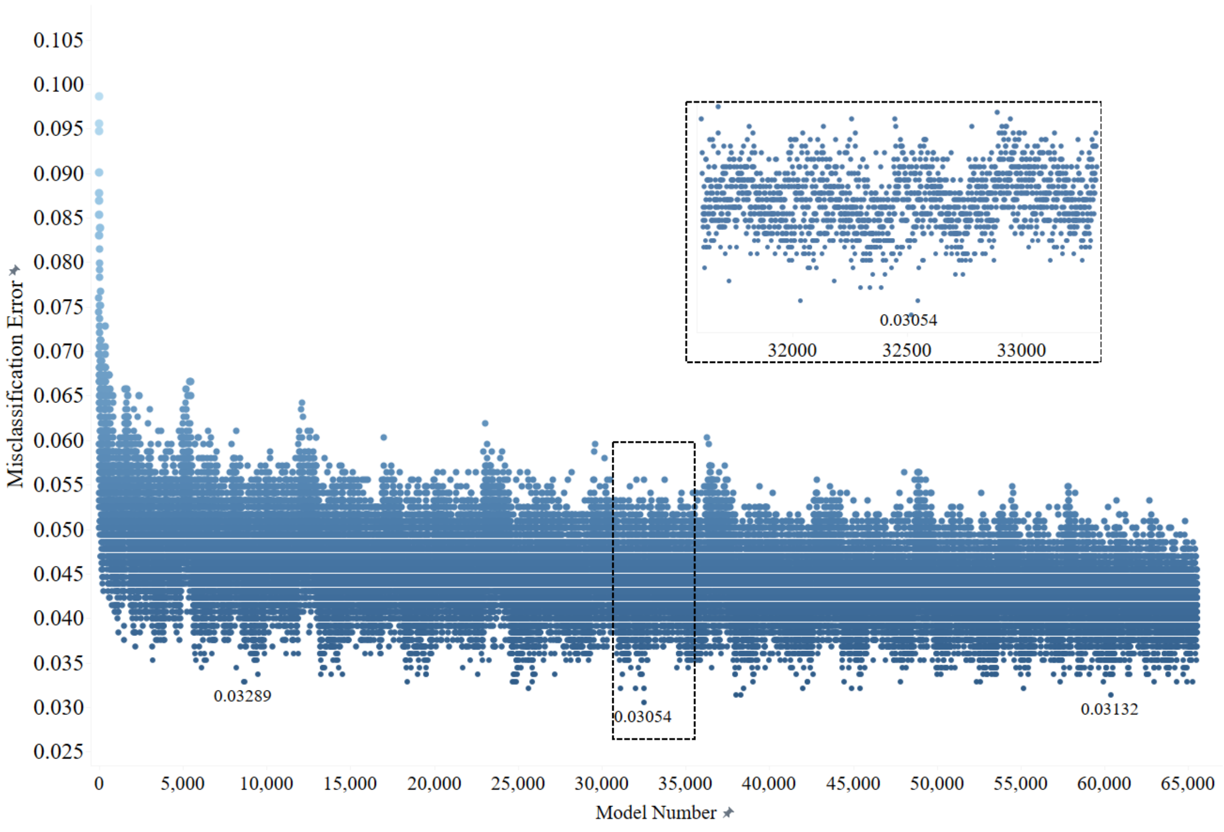

Supplementary Figure 1: Total misclassification error for all possible GCM combinations for RCP 6.0

Supplementary Note 2: Choosing the Optimum Machine Learning Algorithm for the Analysis.

Multiple ML models were developed for predicting the future projection for the Vulnerability categories. The first model is Gradient Boosted Random Forest (RF) model, with a splitting of 70% to 30% between training and testing, respectively. The model performance is optimized through the average Out-of-Bag error (OOB). As shown in Supplementary Figure 2, where the number of trees is set between 1 and 3000 (only showing the first 1000 in Figure 5a), a step size of 1, and values of error calculated for each category separately to identify outlying behavior in the model's performance. The model shows no clear improvement in performance beyond n_{tree} of

500 in RCP 6.0, and slight fluctuations in RCP 8.5 till n_{tree} = 1000. The optimal number of trees in both models was taken at 500 and 1000 for RCP 6.0 and RCP 8.5, respectively.

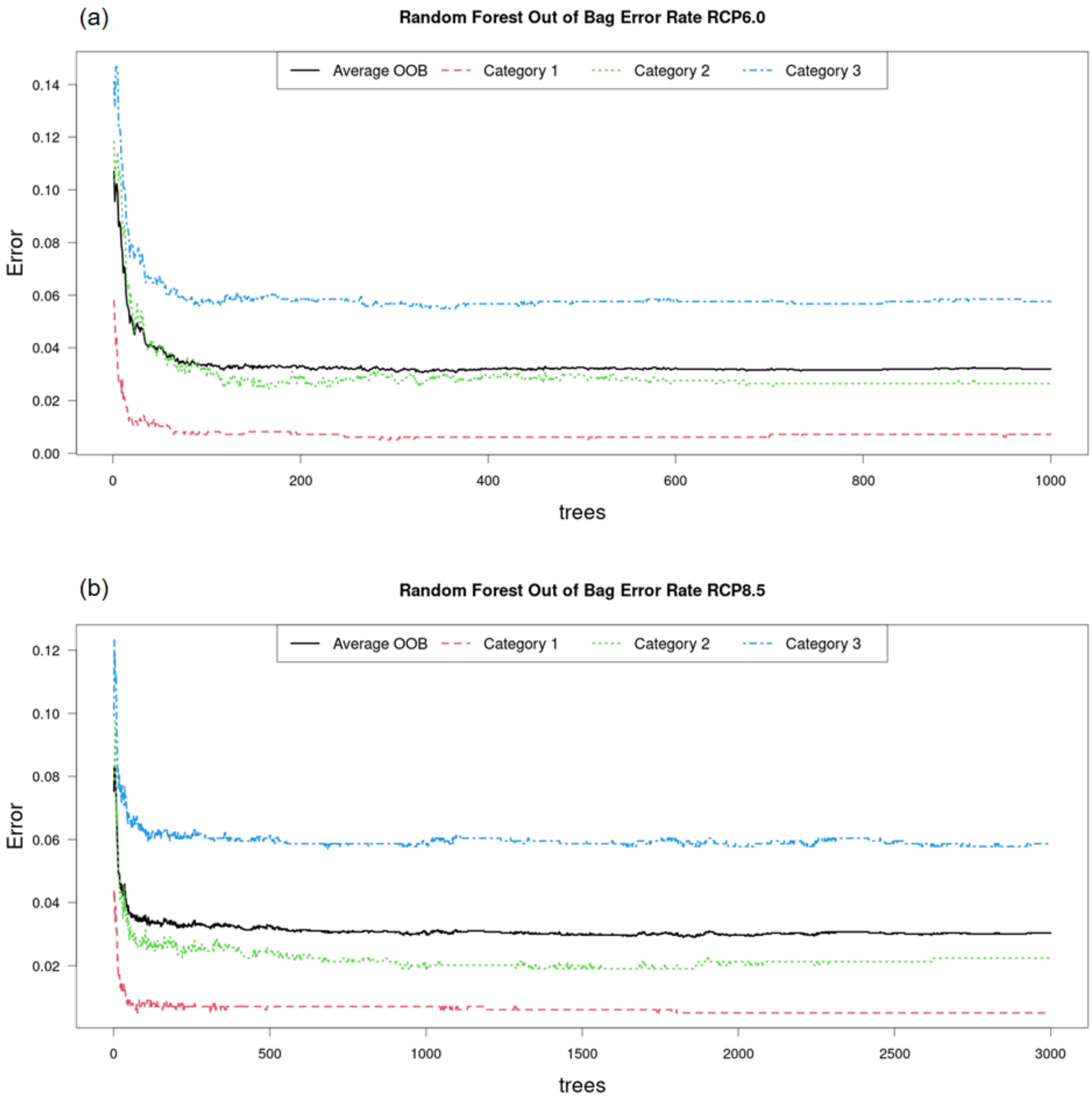

Supplementary Figure 2: Out of Bag error for Random Forest prediction model where: a) for RCP 6.0, and b) for RCP 8.5

The second model is Bagged Decision Tree (DT) with 15,000 bootstrap replication, and 4 random splitting for variables at each split. The model's results were analyzed with the performance measures included in the main manuscript. Supplementary Figures 3 and 4 show the visualization for the performance measures based on the F1-Score, Precision, and Recall (Sensitivity), with their

calculations using [Eq. (5)] through [Eq. (7)] in the main manuscript. This analysis shows us that although the prediction performance of the models is comparable in both, training and testing datasets, the Bagged Decision Tree model slightly outperforms the Random Forest model, however the performance of both models are achieving high efficacy with almost all measures above the 90% mark. Supplementary Figure 3 shows the performance measures for the emission scenario RCP 6.0 for the training and testing datasets, where the Sensitivity, Precision, and F1-Score shown for all categories. Looking at the results of these performance measures, it is clear that the validation of the model performance and prediction objective are high, ensuring that the features included in the analysis yield favorable results in both training and testing datasets. By looking at the training dataset on its own, it can be assumed that the model is overtrained, however, the testing and cross-validation conducted on the dataset also yielded over 90% accuracy and efficacy, ensuring that the model does perform well for future projections. Supplementary Figure 4, on the other hand, show that the performance measures for the emission scenario RCP 8.5 where the Sensitivity, Precision, and F1-Score are shown for all categories. Similar to RCP 6.0, the RCP 8.5 model's performance measures are high with accuracy and efficacy above 90% in all categories, showing similar behavior in the testing category, showing that the prediction threshold is met in both models, albeit with a slight advantage to the performance of the Bagged DT model over the Boosted RF model in both emission scenarios. As such, moving forward, the bagged DT model is chosen for further analysis, interpretability, and future climate projections throughout this study.

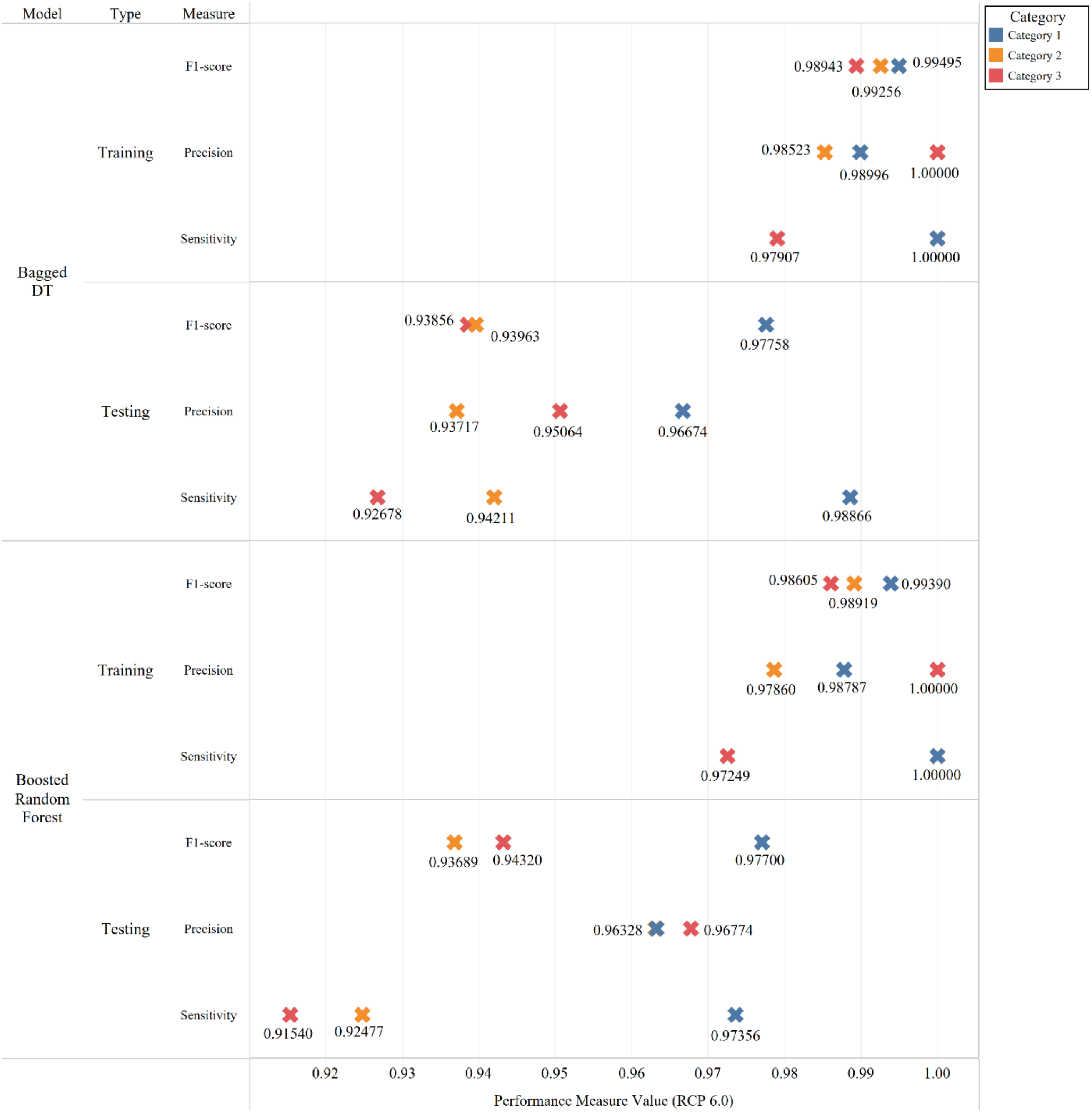

Supplementary Figure 3: Model performance indicators for RCP 6.0

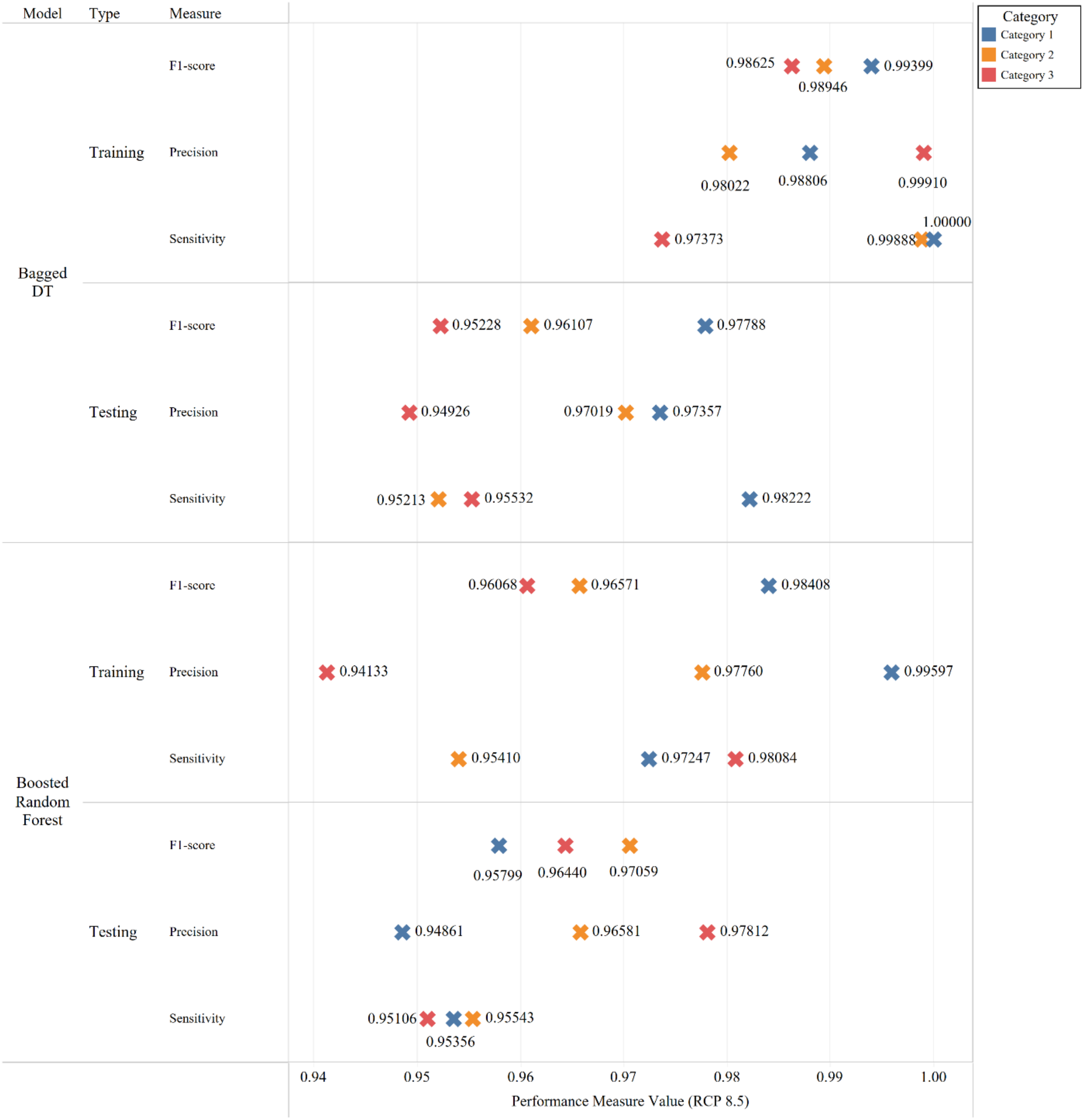

Supplementary Figure 4: Model performance indicators for RCP 6.0

Supplementary Figures 5 and 6 give deeper insight into the performance of the bagged DT model by showing the confusion matrices for the RCP 6.0 and RCP 8.5 respectively. Where (a) is for the training dataset and (b) is for the testing dataset in both figures. From Supplementary Figure 5 we can see that the accuracy of the model is (99.2%) for training dataset, and (95.2%) for testing dataset, showing a misclassification error of (4.7%) for the testing dataset based on [Eq. (4)] in the main manuscript. While Supplementary Figure 6 shows that the accuracy of the training dataset is

(98.8%), and (96.3%) for the testing dataset, showing a misclassification error of just (3.6%) based on [Eq. (4)] in the main manuscript, proving to provide reliable projections for future resilience analysis and planning.

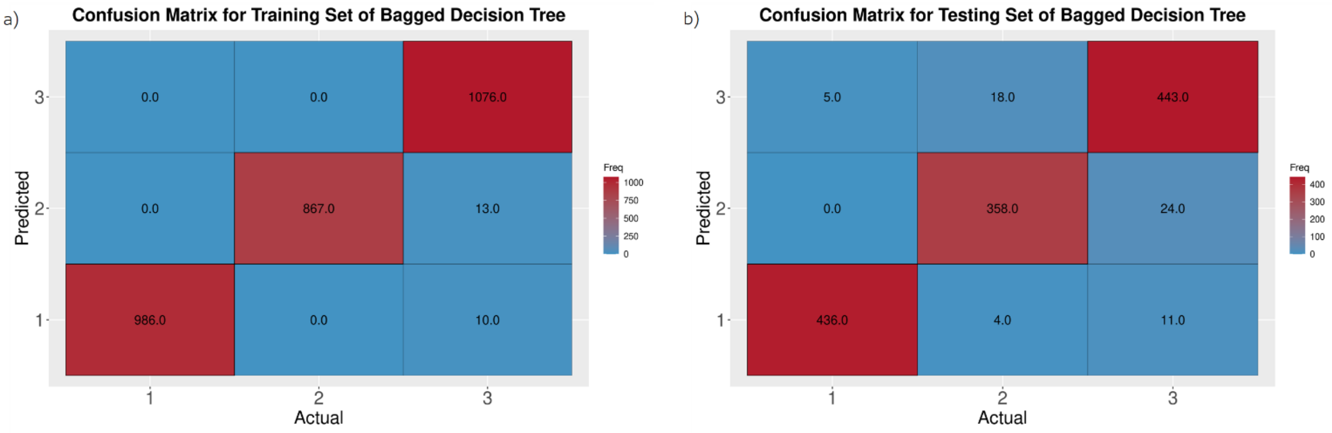

Supplementary Figure 5: Confusion matrix for bagged DT model RCP 6.0 where (a) is training dataset, and(b) is the testing dataset

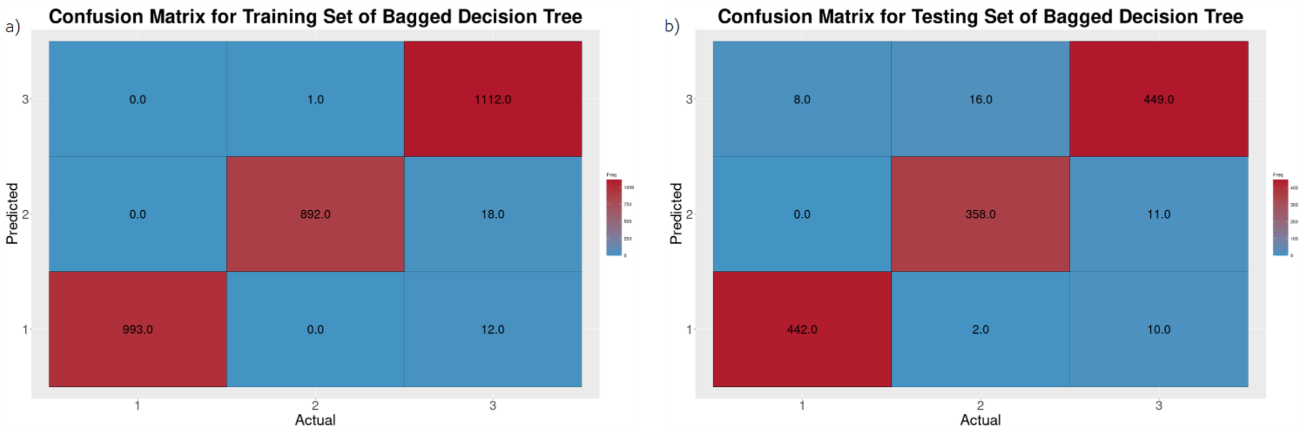

Supplementary Figure 6: Confusion matrix for bagged DT model RCP 8.5 where (a) is training dataset, and(b) is the testing dataset.

Supplementary Note 3: Interpretability of the RCP 6.0 Prediction Model.

The correlation matrix include the correlation value between the input pairs considered in this study. It can be observed that the Temperature variables are highly correlated across different GCMs, however, it can also be observed that the temperature is inversely correlated to wind speed and runoff, but slightly correlated with precipitation. It can be concluded that the wind variables are not correlated with the precipitation and runoff, neither positively or inversely correlated,

however, the precipitation and runoff variables are positively correlated for each GCM simulation, but not across different models, highlighting the need for an ensemble technique for including as many GCMs as possible to expand the range of variables in the analysis and their impact on the prediction models.

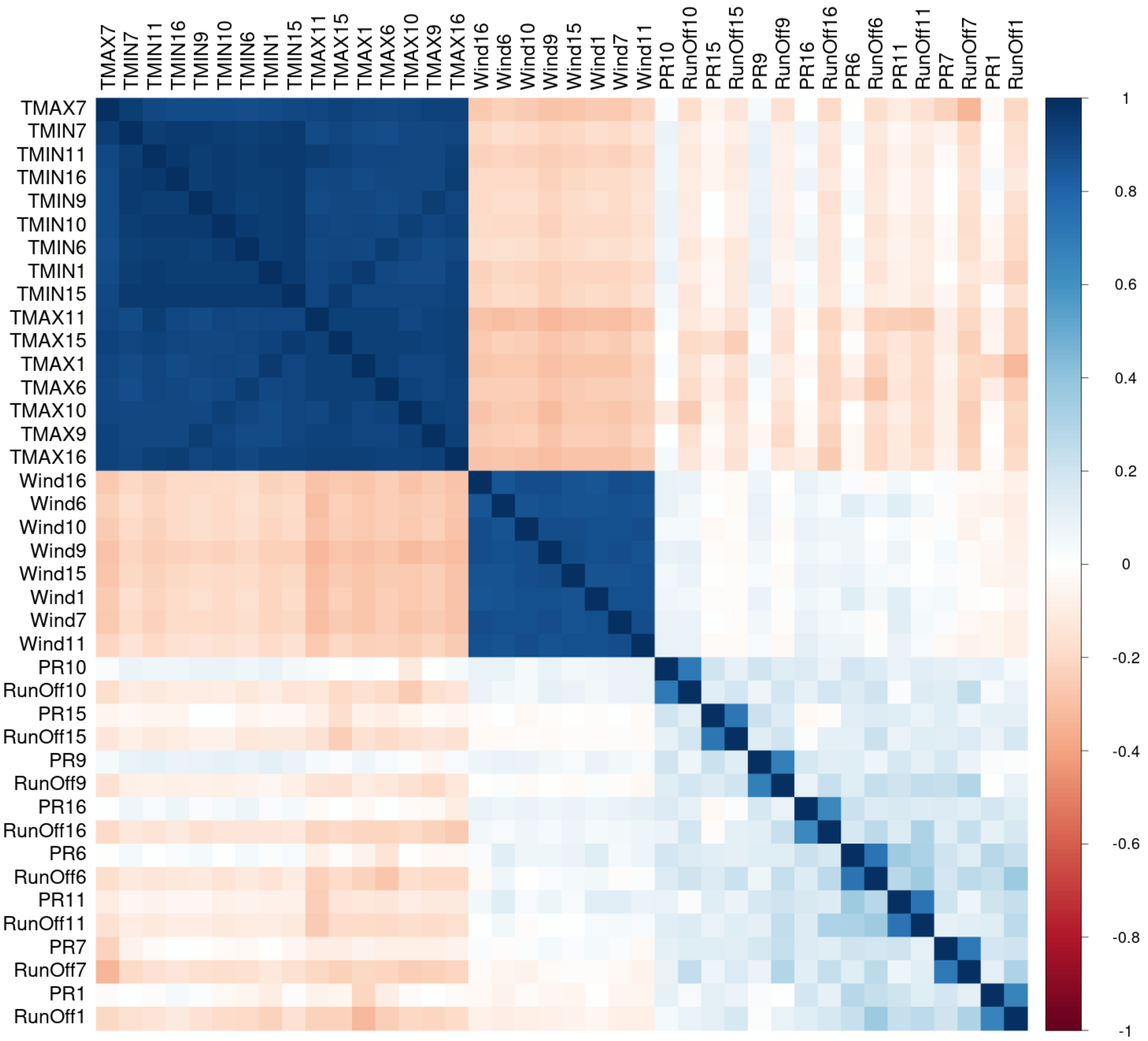

Supplementary Figure 7: Correlation matrix for Bagged DT model RCP 6.0 for the included variables in the prediction.

The following analysis is identifying the Variable Importance (VI) in the model. The VI included in the analysis employs the Receiver Operating Characteristics (ROC) Curve analysis conducted on each predictor. The ROC demonstrates the model's susceptibility to incorrectly classifying the observations in the dataset, where a series of cutoff methods are applied in the predictors for the

for the prediction to take place. The area of the ROC curve is then calculated for each class pair (i.e., Category 1 vs Category 2, Category 2 vs Category 3, etc.) using the trapezoidal rule, then the maximum area under the curve across the relevant pair-wise curves is considered the VI in the model[1]. From Supplementary Figure 8, we can conclude that the first 11 variables are maximum and minimum temperatures from various GCMs, which is confirmed by the high correlation of temperature variables with other variables in the dataset. It is also clear that after a certain threshold, the VI drops significantly, indicating that some GCMs have higher influence over the probability of correctly classifying a class.

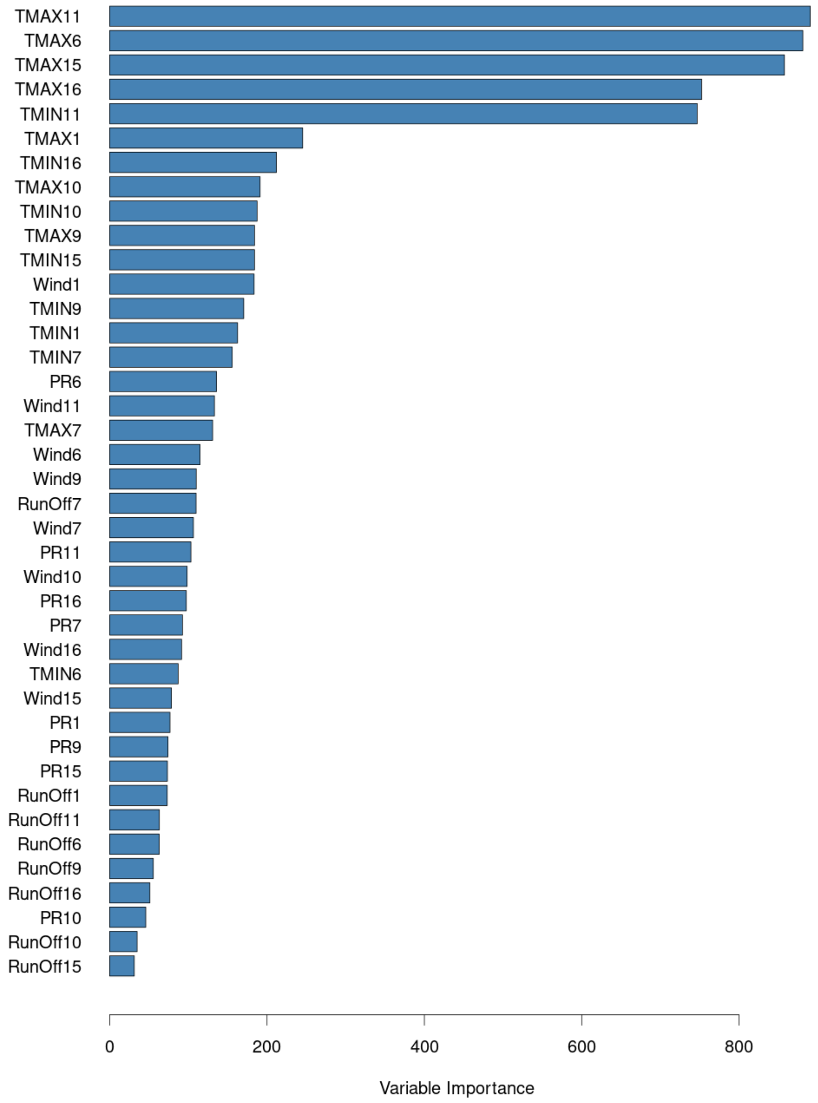

Supplementary Figure 8: Variable Importance for the included variables in bagged DT model for RCP 6.0

The most essential interpretability technique is the Partial Dependence Plot. Details on how that is calculated can be found in the main manuscript. Supplementary Figure 9 shows the first four maximum temperature variables, the first two minimum temperatures, the first two precipitation variables, the first two wind speed variables, and the first two runoff variables based on their importance according to the VI plots for RCP 6.0. The values of the variables in the model are normalized and scaled for an optimum homogeneity in the ML model, to avoid skewness in the model and account for the multiple different units of measurement for all variables, it was unscaled back to its original range for the development of the PDPs, to draw interpretability insights. While it is expected that the results will vary depending on the GCM model results being inspected within the input space of the ML model and the emission scenario under investigation, the overall behavior of these variables is expected to remain the same. The PDPs for the maximum temperatures all indicate a clear jump in the influence of the vulnerability category when the maximum temperatures are between 30 to 40 ^{o}C, showing that the risk of flooding disasters increase as the temperatures rise, the same applies for all GCMs included in the development of the predictive model. The minimum temperatures do not have an equivalent increase in predicted flood risk impact, but it shows a slight rise when monthly minimum temperature is between 15 to 30 ^{o}C. this can be attributed to the increased heat content over the gulf of Mexico, transforming the weather into a tropical atmosphere, positively correlated to increased rainfall and a suitable climate for the development of hurricanes[2,3]. The runoff doesn't have much impact on the predictive capability of the model beyond the 100 mm, however, the impact is slightly different from one GCM to another from 0 to 100 mm. The vulnerability category is higher at lower runoffs, then gradually fluctuates and falls until it reaches 100 mm. The precipitation patterns heavily vary from one GCM to another, however at low values (below the 200 mm), their influence is similar to one another where it keeps fluctuating at lower values. The windspeed's influence over the vulnerability category is very high for values up to 6 (m/s), with a severe drop in influence over predicted vulnerability category increase once the speed goes beyond the 6 m/s threshold.

Partial Dependence Plots for RCP6.0

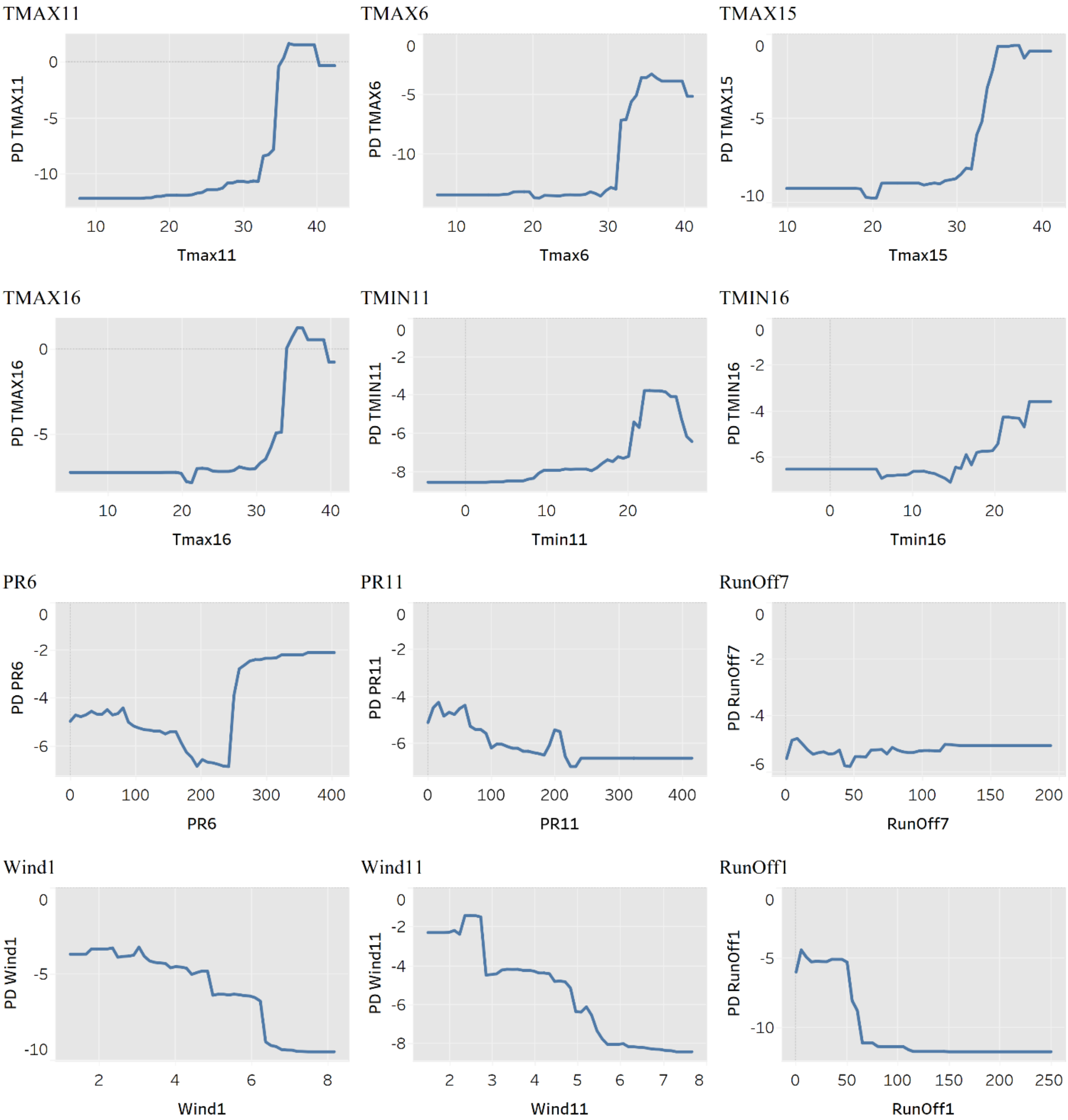

Supplementary Figure 9: Partial Dependence Plots for RCP 6.0

Supplementary Note 4: Results for the RCP 6.0 Prediction Model.

Supplementary Figure 10 a) is a temporal distribution of the yearly average vulnerability category per county per year between the years 2020 and 2050, b) is the spatial distribution of the counties involved in the analysis, *i*) is a multi-layer spatial distribution of the year 2020 where the location of each county is identified by a circle, the size and color of the circle represents the running cumulative average vulnerability category, *ii*) a multi-layer spatial distribution of the running cumulative average till the year 2030, *iii*) a multi-layer spatial distribution of the running cumulative average until the year 2040, iv) a multi-layer spatial distribution of the running cumulative average until the year 2050, also differentiated by size and color for visualization. This analysis shows that there is an increase in the yearly average flood vulnerability index for almost all counties involved in the analysis, amounting to an increase in the yearly average across the years, albeit a small increase. The average category between the years 2020 and 2030 is 2.1, between the years 2030 and 2040 the average is 2.17, and 2.21 between the years 2040 and 2050. The historical recorded damage between the years 1996 and 2020 is $13,686M, amounting to an average of $5,702M per decade. Assuming the historical category is the same as that at the start of 2020, the projected damage based on the RCP 6.0 scenario between the years 2020 and 2030 is $6,260M, at an increase of 9.7% in monetary damage, between the years 2030 and 2040 the projected monetary loss is $6,498M at an increase of 13.9%, and between the years 2040 and 2050, the projected monetary loss is at $6,587M at an increase of 15.5%. Notwithstanding this monetary damage, the vulnerability category is also an indicator for other socio-economic components, like injuries, fatalities, evacuations, and the downtime of the community following the flood event. The indicator shows an increase of 15.5% per decade in these components leading up to the year 2050, showing the immediate need for intervention and mitigation measures and the development of a resilience-guided flood risk policies. These numbers, while alarming, they only amount for the counties and geographical locations included in the analysis presented herein, not the entirety of the state of Texas, which would amount to a much larger overall increase.

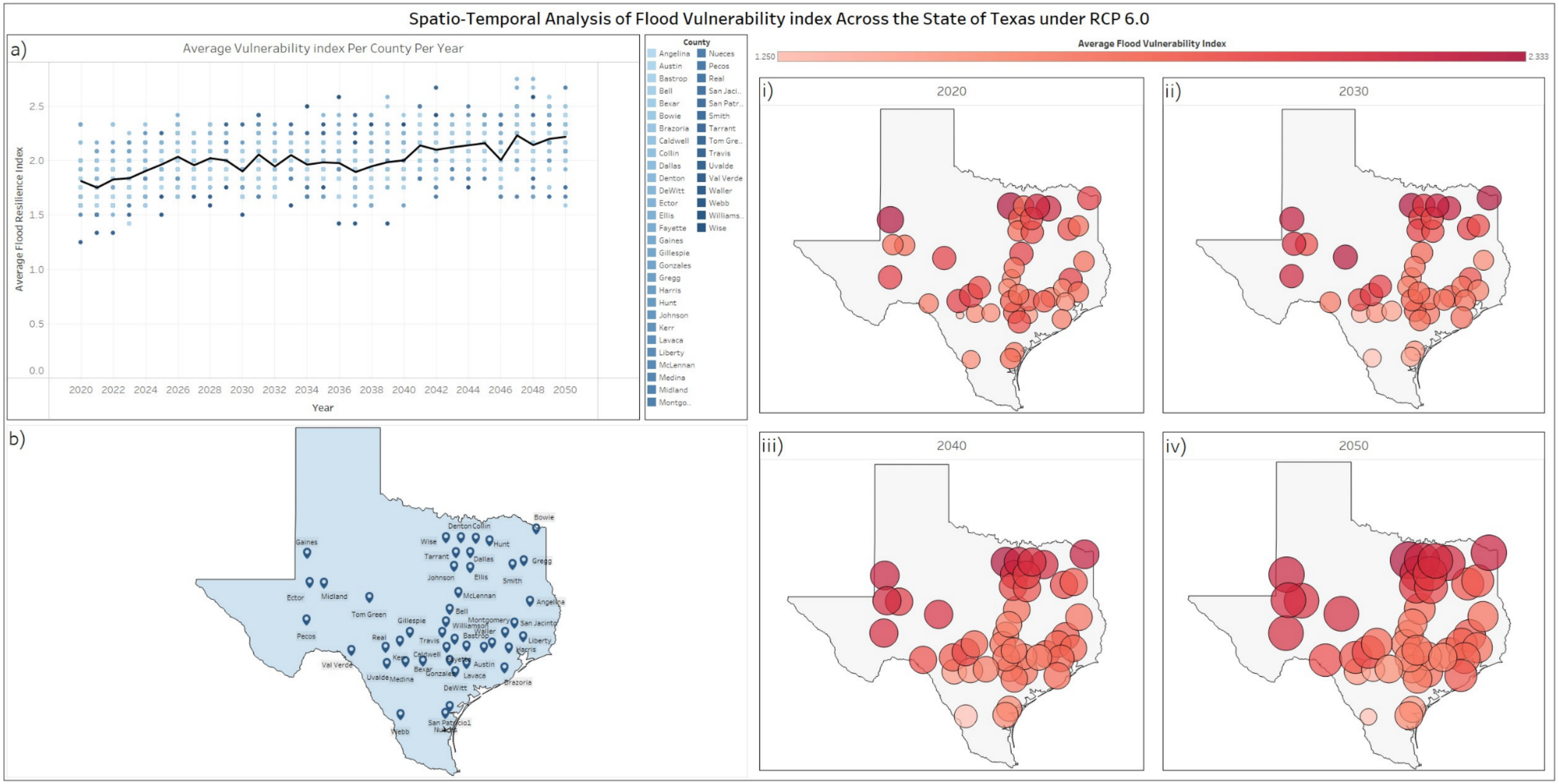

Supplementary Figure 10: Spatial Twin: a Spatio-temporal Model Output visualization for RCP 6.0, where; a) yearly average per county per year, with a running average for all included counties, b) spatial distribution of included counties and their GCM's stations, i) the spatial distribution of average Vulnerability index per county in the year 2020, ii) spatial distribution of average Vulnerability index per county till the year 2030, iii) spatial distribution of average Vulnerability index per county till the year 2040, iv) spatial distribution of average Vulnerability index per county till the year 2050.

Supplementary References

1. Kuhn, M. The caret Package. https://topepo.github.io/caret/index.html (2019).
2. NOAA Office for Coastal Management. Texas. https://coast.noaa.gov/states/texas.html (2021).
3. Perica, S. *et al. Precipitation-Frequency Atlas of the United States Volume 11 Version 2.0: Texas*. vol. 11 (2018).
